# ZIPCO, a putative metal ion transporter, is crucial for *Plasmodium* liver-stage development

**DOI:** 10.15252/emmm.201403868

**Published:** 2014-09-25

**Authors:** Tejram Sahu, Bertrand Boisson, Céline Lacroix, Emmanuel Bischoff, Quentin Richier, Pauline Formaglio, Sabine Thiberge, Irina Dobrescu, Robert Ménard, Patricia Baldacci

**Affiliations:** 1 Institut Pasteur, Unité de Biologie et Génétique du PaludismeParis Cedex 15, France; 2 Laboratory of Malaria Immunology and Vaccinology, National Institute of Allergy and Infectious DiseasesRockville, MD, USA; 3 St. Giles Laboratory of Human Genetics of Infectious Diseases, The Rockefeller UniversityNew York, NY, USA; 4 Institut de Biologie et Chimie des ProtéinesLyon Cedex 07, France; 5 Institut Pasteur, Plateforme Puces à ADN GénopoleParis Cedex 15, France

**Keywords:** iron, liver stage, *Plasmodium*, transporter, ZIP

## Abstract

The malaria parasite, *Plasmodium*, requires iron for growth, but how it imports iron remains unknown. We characterize here a protein that belongs to the ZIP (Zrt-, Irt-like Protein) family of metal ion transport proteins and have named ZIP domain-containing protein (ZIPCO). Inactivation of the ZIPCO-encoding gene in *Plasmodium berghei*, while not affecting the parasite's ability to multiply in mouse blood and to infect mosquitoes, greatly impairs its capacity to develop inside hepatocytes. Iron/zinc supplementation and depletion experiments suggest that ZIPCO is required for parasite utilization of iron and possibly zinc, consistent with its predicted function as a metal transporter. This is the first report of a ZIP protein having a crucial role in *Plasmodium* liver-stage development, as well as the first metal ion transporter identified in *Plasmodium* pre-erythrocytic stages. Because of the drastic dependence on iron of *Plasmodium* growth, ZIPCO and related proteins might constitute attractive drug targets to fight against malaria.

## Introduction

Iron is essential for the growth of organisms ranging from bacteria to mammals. During an infection, pathogen and host compete for iron and the outcome impacts disease severity. Pathogens have evolved efficient tools to scavenge iron, and iron uptake ability is linked to virulence in a broad range of bacteria, protozoa and fungi (Schaible & Kaufmann, 2004; Schrettl *et al*, 2004; Crouch *et al*, 2008; Seifert *et al*, 2008). The host controls iron availability as a defence mechanism against invading pathogens, termed nutritional immunity (Cassat & Skaar, 2013). A master regulator of iron metabolism and redistribution is the peptide hormone hepcidin. Induced upon infection or inflammation, hepcidin depletes iron from the plasma by binding to ferroportin thereby decreasing absorption from the intestine, recycling from macrophages and release of iron stored in hepatocytes (Ganz, 2011; Drakesmith & Prentice, 2012).

During malaria, the *Plasmodium* parasite undergoes several cycles of multiplication in the host, in both hepatocytes and red blood cells (RBC). The parasite form (sporozoite) injected by an infected mosquito invades a hepatocyte, where it transforms into tens of thousands of hepatic merozoites, the RBC-infective forms (Graewe *et al*, 2012). Once released into the blood, these forms each invade a RBC and multiply into 10–30 new parasites, initiating the successive cycles of parasite replication in the blood. While the brief liver phase of infection remains asymptomatic, the blood phase of infection causes the symptoms of the disease.

Iron availability is known to influence the clinical expression of malaria infection. Many clinical trials have concluded that iron deficiency decreased susceptibility to malaria while iron supplementation increased blood-stage parasitaemia and disease pathology (Murray *et al*, 1978; Kabyemela *et al*, 2008; Gara *et al*, 2010). In particular, a large study in Pemba on over 24,000 children demonstrated that prophylactic supplementation with iron and folic acid in a population with high rates of malaria resulted in an increased frequency of severe disease and death (Sazawal *et al*, 2006; Prentice *et al*, 2007). The Fe^3+^ chelator desferrioxamine (DFO) inhibits the growth of *P. falciparum* in RBC *in vitro*, causing a block in schizogony (Raventos-Suarez *et al*, 1982). DFO also reduces blood-stage parasitaemia in mice (Fritsch *et al*, 1985; Hershko *et al*, 1992), primates (Pollack *et al*, 1987) and humans, where it was shown to ameliorate cerebral malaria in children (Gordeuk *et al*, 1992). The liver stage of *Plasmodium* also requires iron for growth. *In vitro*, DFO-mediated iron depletion was found to inhibit schizogony of both *P. yoelii* and *P. falciparum* in rodent and human hepatocyte cultures, respectively (Stahel *et al*, 1988).

Little is known of how *Plasmodium* scavenges iron and no specific iron transporter has been described in *Plasmodium* so far. Here, we demonstrate that a Zrt- Irt- Like protein in *P. berghei* is specifically important for the growth and development of the parasite liver stage, and we provide evidence suggesting that this protein acts as an iron, and probably zinc, transporter.

## Results

### Identification of *ZIPCO*

We identified the gene PBANKA_050650 in a previous screen for *P. berghei* genes displaying an increased expression during pre-erythrocytic stages of the parasite compared to parasite red blood cell (RBC) stages (Ishino *et al*, 2009; Boisson *et al*, 2011). Quantitative RT-PCR analysis of various *P. berghei* developmental stages indicated that PBANKA_050650 transcripts were sevenfold higher in sporozoites than mixed RBC or exo-erythrocytic forms (EEFs) developing inside HepG2 hepatoma cells (Supplementary Fig S1, *P* = 0.0078). The cDNA of PBANKA_050650 was cloned from *P. berghei* infected HepG2 cells, and its sequence was found to be identical to that present in GenDB and PlasmoDB (version 10.0).

**Figure 1:**
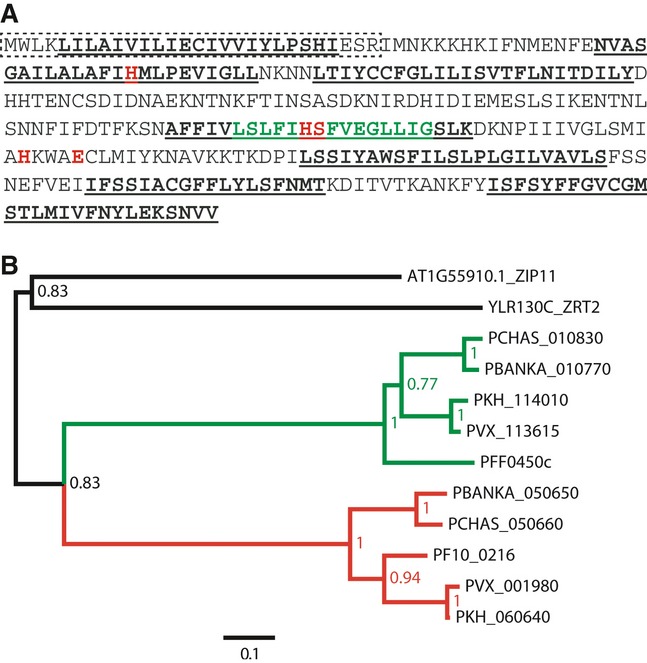
ZIPCO belongs to the ZIP family of transport proteins Protein sequence of ZIPCO. The signal peptide, predicted with SignalP 3.0 Server, is indicated by the box with dotted line; the consensus trans-membrane domains, as predicted with TMHMM server v 2.0, TMPred Server, DAS and Plasmo DB annotation, are indicated by underlined bold letters; the ZIP signature sequence is indicated by green letters; conserved residues that have been shown to be important for transport of metal ions by IRT1 and LIT1 (Rogers *et al*, 2000; Jacques *et al*, 2010) are shown in bold red letters.Phylogenetic tree showing evolutionary distance between *P. berghei* ZIPCO and other *Plasmodium* species. The *S. cerevisae* protein ZRT2 and the *A. thaliana* protein ZIP11 were used as an out-group. The bootstrap values are indicated on each branch.

PBANKA_050650 is predicted to code for a protein with seven trans-membrane (TM) domains, including a putative N-terminal signal peptide (Fig 1A), and has been annotated as a putative zinc transporter due to the presence of a conserved ZIP domain (Pfam02535). The ZIP family of proteins have a conserved signature of 15 amino acids, located either completely or partially within the fourth TM region (Eng *et al*, 1998), and 13 of them are present in PBANKA_050650 (Fig 1A). Sequence comparison using the forty sequences that constitute the seed of the Pfam02535 profile found that the well-conserved amino acids of this profile are present in PBANKA_050650 (Supplementary Fig S2). The genomes of *P. falciparum, P. chabaudi, P. knowlesi, P. vivax, Saccharomyces cerevisiae* and *Arabidopsis thaliana* were screened with the Pfam02535 profile, and a phylogenetic tree was generated (Fig 1B) that indicated the presence of two paralogs of the ZIP family in the screened *Plasmodium* species. In *P. berghei*, the paralog of PBANKA_050650 is PBANKA_010770, annotated as a putative permease. PBANKA_050650 thus encodes a protein of the ZIP family, which we named ZIP domain-containing protein, ZIPCO.

### ZIPCO is not essential for *P. berghei* blood-stage growth

**Figure 2:**
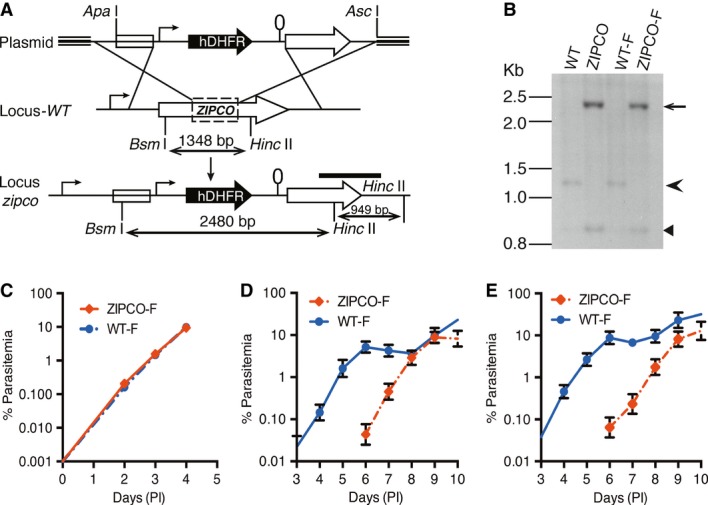
ZIPCO-F parasites develop normally in blood stages but sporozoites are less infectious to the mammalian host Schematic representation of *P. berghei zipco* targeted gene disruption. The plasmid pBC-*zipco*-hDHFR used for homologous recombination is shown on the top with the hDHFR cassette indicated by a black arrow. The endogenous locus (Locus*-WT*) is shown in the middle; the dotted rectangle indicates the region deleted and replaced by the hDHFR cassette. The resulting recombinant locus is shown in the lower part. Sizes of restriction fragments obtained after digestion of genomic DNA with *Bsm*I and *Hinc*II are indicated by double-sided arrows. The probe used for Southern blot analysis (see panel 2B) is shown as a black bar.Analysis of *zipco* locus in WT and recombinant parasites. Genomic DNAs from NK65 (WT), ANKA-GFP (WT-F), and recombinant clones ZIPCO and ZIPCO-F were digested with *Bsm*I and *Hinc*II. A probe from the 3′UTR region (shown in 2A) was used for analysis. The WT (1,348 bp) and recombinant (2,480 bp) restriction fragments detected are indicated by an arrowhead and arrow, respectively. The 949 bp *Hinc*II fragment, present in both WT and recombinant loci, is indicated by a small arrowhead.Graph showing the multiplication of WT-F and ZIPCO-F blood stages. Mice were injected with mixed blood stages to obtain an initial parasitemia of 0.001%. The parasitemia was followed daily for 4 days on Geimsa-stained blood smears. Graph represents the mean parasitaemia ± SD (*n* = 6 mice in each group).Graph showing the mean parasitemia ± SD of mice after intradermal injection with 25,000 WT-F or ZIPCO-F sporozoites. (*n* = 5 mice in each group).Graph showing the mean parasitemia ± SD of mice after intravenous injection with 25,000 WT-F or ZIPCO-F sporozoites. (*n* = 5 mice in each group). Source data are available online for this figure.

The *ZIPCO* locus was disrupted by double crossover homologous recombination in two strains of *P. berghei,* an ANKA line expressing GFP (Janse *et al*, 2006a) and NK65, named herein as WT-F and WT, respectively. A 540 bp-long internal fragment of *ZIPCO* encoding the fourth TM region, including the ZIP signature sequence and part of the fifth TM region of the protein, was deleted and replaced by the hDHFR selectable cassette (Fig 2A). Recombinant clones obtained by limiting dilution, named ZIPCO-F (ANKA) or ZIPCO (NK-65), were found by Southern blot analysis (Fig 2B) to have the expected *ZIPCO* recombinant locus. Counting parasitemia (proportion of parasite-harbouring erythrocytes) on Giemsa-stained blood smears or by cytometry showed that WT-F and ZIPCO-F parasites had similar multiplication rates during parasite exponential growth in the blood of mice (Fig 2C). This result indicates that ZIPCO is not essential for parasite multiplication inside erythrocytes.

### ZIPCO is important for EEF development

Mice infected with WT-F or ZIPCO-F parasites were then used to infect *A. stephensi* mosquitoes. Similar numbers of WT-F and ZIPCO-F parasites sporozoites were counted in mosquito salivary glands 21–25 days after parasite transmission (Supplementary Table S1). Equal numbers of WT-F or ZIPCO-F salivary gland sporozoites were injected into mice intradermally (ID) or intravenously (IV), and pre-patent periods of infection were assessed by Giemsa-stained blood smears. ZIPCO-F blood-stage parasites emerged with a 3–4 day delay compared to WT-F, independently of the sporozoite injection route (Fig 2D–E). Since the multiplication in the blood stages is normal, these results indicated a approximately 10^3^ loss in infectivity for the mutant during pre-erythrocytic development. A similar delay was observed with ZIPCO sporozoites after IV injection (Supplementary Table S2).

**Figure 3:**
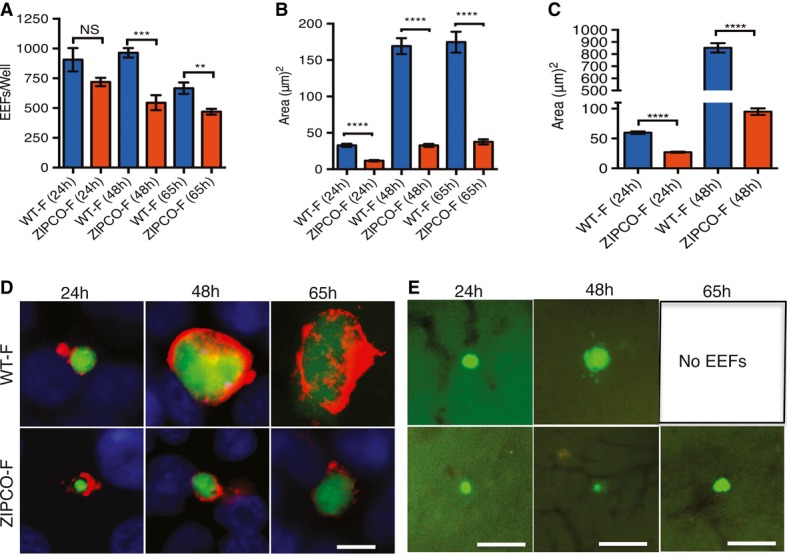
ZIPCO-F liver stages have severe growth defect *in vitro* and *in vivo* Histograms of the number of exo-erythrocytic parasites (EEFs). HepG2 cells were infected with WT-F or ZIPCO-F salivary gland sporozoites and at 24, 48 and 65 h postinfection the developing parasites were detected with either anti-UIS4 antibody (24 h), or anti-EXP-1 antibody (48 and 65 h) as shown in panel D. Data represent mean ± SEM of two experiments, one performed in triplicate and one in duplicate. NS means not significant; ***P = 0.0005, **P = 0.0064.Histograms of the size WT-F and ZIPCO-F EEFs *in vitro* at 24, 48 and 65 h postinfection. (WT-F *n* = 19, 53 and 27 at 24, 48 and 65 h, respectively; ZIPCO-F *n* = 21, 36 and 25, respectively). The values are mean area with ± SEM, ****P < 0.0001. Images were acquired on Zeiss epiflourescence microscope and analysed by ImageJ software.Histograms of the size of WT-F and ZIPCO-F parasites *in vivo*. Values represent the mean area ± SEM at 24 and 48 h postinfection. (*n* = 27 for both parasites and both time points). ****P < 0.0001.Representative images of WT-F and ZIPCO-F EEFs *in vitro*. EEFs are green and labelling with anti-UIS4 or anti-EXP-1 is shown in red. Note the small size of the ZIPCO-F compared to WT-GFP parasites. Scale bar represents 10 μm. Images were acquired on Zeiss epiflourescence microscope and analysed by ImageJ software.Representative images of WT-F and ZIPCO-F liver stages *in vivo*. C57Bl/6j mice were injected with 150,000 WT-F or ZIPCO-F salivary gland sporozoites, and 24, 48 and 65 h postinfection livers were isolated and fluorescent microscopy used to detect parasite development. Parasites are shown in green. At 65 h postinfection, no WT EEFs were observed. Scale bar represents 31 μm in 24 h time point and 63 μm in 48 h and 65 h time point.

When examined *in vitro*, WT-F and ZIPCO-F sporozoites had similar gliding capacities on glass slides (Supplementary Fig S3A), and no differences were observed in their ability to traverse and invade host cells *in vitro* (Supplementary Fig S3B). EEF development was then studied *in vitro* using HepG2 cells. Although similar numbers of WT-F and ZIPCO-F EEFs were counted by fluorescence microscopy at 24 hpi (Fig 3A), significantly fewer ZIPCO-F than WT-F EEFs were observed at 48 hpi (43%, *P* = 0.0005) and 65 hpi (30%, *P* = 0.0064). Most notably, the average size of ZIPCO-F EEFs was significantly smaller at all time points (Fig 3B and D), being approximately 65, 80 and 78% that of WT-F EEFs at 24, 48 and 65 hpi, respectively (*P* < 0.0001, Fig 3B). This decrease in size was also observed in the EEFs of the recombinant clone ZIPCO (Supplementary Fig S4A). EEF development was also examined by intra-vital fluorescence microscopy at 24, 48 and 65 after IV of sporozoites (Fig 3C and E). Like *in vitro*, ZIPCO-F EEFs were significantly smaller than the WT-F (*P* < 0.0001; Fig 3C), displaying a approximately 90% reduction in size at 48 hpi. At 65 hpi, WT-F EEFs were no longer observed, having liberated merozoites, while a few ZIPCO-F EEFs were still detected. These data indicated that mutant EEFs were affected both in growth and viability, resulting in reduced and delayed release of merozoites into the blood.

To quantify the mutant defect, HepG2 cells were incubated with equal numbers of GFP^+^ ZIPCO-F or RFP^+^ L733 (Sturm *et al*, 2009) sporozoites, and the number of merozoites of each type released from merosomes at 66 and 90 hpi were counted by fluorescence microscopy. At 66 hpi, at the peak of merosome release in the WT-F EEFs, approximately 1,900 times fewer ZIPCO-F than control merozoites were counted (1.37 × 10^6^ RFP^+^, 720 GFP^+^), in agreement with the approximately 3 day delay in the onset of detectable blood-stage infection in mice infected with ZIPCO-F parasites. At 90 hpi, only approximately 16-fold fewer ZIPCO-F than control merozoites were counted, due to the drastic reduction in merozoite release by the WT at this time (29,700 RFP^+^, 1,800 GFP^+^). Importantly, staining of infected HepG2 cells with DAPI showed that ZIPCO-F EEFs had only a small number of nuclei, including at late time points, in contrast to the numerous nuclei in WT-F EEFs (Supplementary Fig S4B). Therefore, ZIPCO-F parasites undergo a delayed and inefficient schizogonic process in the hepatocyte.

### ZIPCO production peaks in late liver stages

To address ZIPCO protein expression and subcellular localization, we generated by allelic exchange, in the WT-F background, the recombinant ZIPCO-HA parasite in which a HA tag was fused in frame to the carboxy terminus of ZIPCO (Supplementary Fig S5A and B). The ZIPCO-HA clone infected mosquitoes to the same degree as WT (Supplementary Table S1), and the sporozoites were normally infectious to mice (Supplementary Table S2) indicating that the ZIPCO-HA-encoding gene, which replaced WT *ZIPCO*, was fully functional.

To assess the expression of ZIPCO-HA in sporozoites, we performed a Western blot using anti-HA monoclonal antibodies. As shown in Supplementary Fig S5C, we were unable to detect a specific band at the expected 35 kDa in ZIPCO-HA sporozoites.

**Figure 4:**
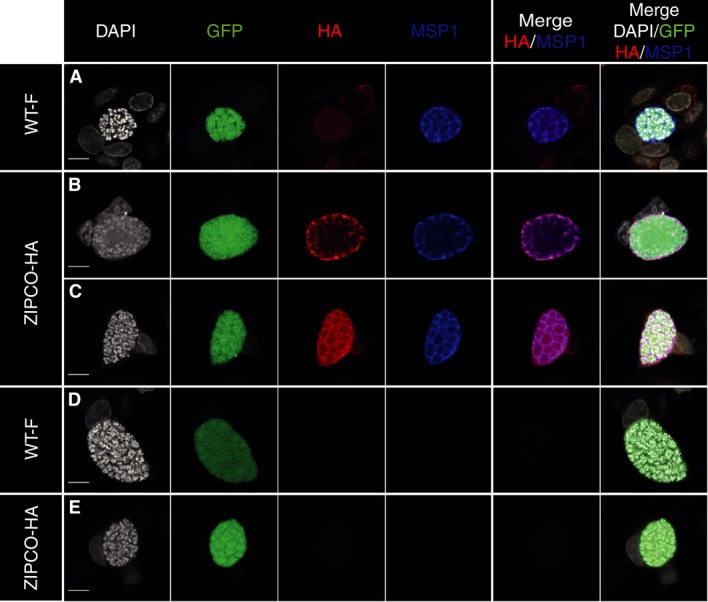
ZIPCO-HA is expressed during late liver stages Indirect immunofluorescence assays performed on HepG2 cells 48 h postinfection with WT-F (panels A and D) or ZIPCO-HA sporozoites (Panels B, C and E). Left column, nuclei detected by DAPI labelling shown in white; second column detection of GFP-expressing EEFs (green); third column panel A, B and C: labelling obtained with anti-HA antibody detected with secondary antibody-AF568 shown in red; fourth column labelling of the plasma membrane with anti-MSP1 antibody detected with secondary antibody-AF647 shown in blue. Columns on right show merged images. Panels D and E show the labelling obtained with secondary antibodies alone. Scale bar represents 10 μm.

To investigate ZIPCO-HA production by and localization in liver stages, ZIPCO-HA infected HepG2 cells were stained using a monoclonal anti-HA antibody. A specific signal was detected in EEFs at 48 and 63 hpi (Fig 4 and Supplementary Fig S5D). At 48 hpi, there was a punctuated labelling within and at the periphery of the developing parasite (Fig 4B). Double labelling with MSP-1, a parasite protein located at the plasma membrane, showed regions of overlap with the HA signal (Fig 4B merged image). In more advanced EEFs at the cytomere stage (Graewe *et al*, 2011), the HA labelling was detected around groups of nuclei and again there was overlap with the MSP1 signal (Fig 4C). These data strongly suggest that ZIPCO-HA is mainly located at the parasite plasma membrane.

### *ZIPCO* mutant deficiency is reversed by iron and zinc

**Figure 5:**
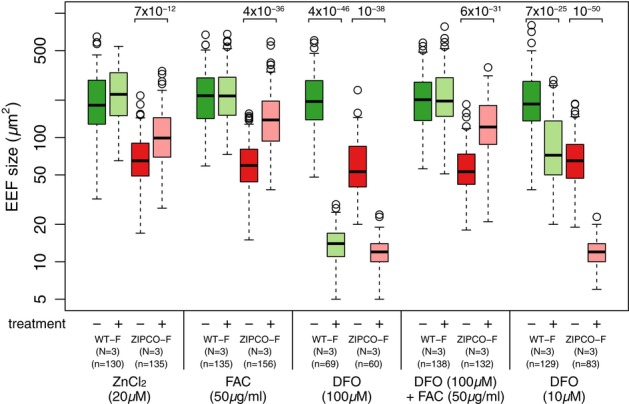
Supplementation with iron or zinc increases the size of ZIPCO-F EEFs HepG2 cells were infected with either WT-F or ZIPCO-F sporozoites and cultured in DMEM supplemented with 20 μM zinc chloride (ZnCl_2_); 50 μg/ml ferric ammonium citrate (FAC); Deferoxamine mesylate salt (DFO) 10 or 100 μM; DFO 100 μM and FAC 50 μg/ml. Images were taken at 46 h postinfection, and the areas were calculated with ImageJ software. Data were obtained from three independent experiments (see Supplementary Fig S6). Sizes of WT-F EEFs in DMEM are shown as dark green boxes; pale green shows size of WT-F EEFs in different media; ZIPCO-F EEFs in DMEM are shown in red and in pink for other media. (−) indicates DMEM alone; (+) indicates supplemented media; N is the number of replicates; n is the number of EEFs measured per treatment. Significant *P*-values are indicated; refer to Supplementary Table S4 for complete statistical analysis.

To determine whether ZIPCO might be transporting zinc and/or iron, WT-F and ZIPCO-F EEFs were first grown in media supplemented with 20 μM zinc chloride (ZnCl_2_). This treatment had no significant effect on the size of WT EEFs (*P* = 0.1 Supplementary Table S4), but significantly increased the size of mutant EEFs at 46 hpi (*P* = 7.1 × 10^−12^ Fig 5, Supplementary Fig S6 and Supplementary Table S4). This suggested that the mutant growth defect was partly due to zinc deficiency. To further test the role of zinc in EEF growth, we allowed EEFs to grow in the presence of TPEN, a zinc chelator. However, at 2 μM concentration, TPEN yielded inconsistent results, while higher concentrations of TPEN were toxic for host cells (not shown).

We then tested the role of iron in EEF development. We first asked whether ferric ammonium citrate (FAC) might revert the mutant defect. HepG2 cells were infected with WT-F and ZIPCO-F sporozoites, fresh medium with 50 μg/ml FAC was added at 2 hpi and the sizes of EEFs were measured at 46 hpi. Addition of FAC did not significantly increase the size of WT-F EEFs at 46 hpi (*P* = 0.12, Fig 5, Supplementary Fig S6 and Supplementary Table S4), showing that iron was not limiting in our experimental conditions. In contrast, addition of FAC significantly increased the size of ZIPCO-F EEFs (*P* = 3.6 × 10^−36^, Fig 5, Supplementary Fig S6 and Supplementary Table S4). This showed that the mutant growth defect was at least partly due to iron deficiency and that ZIPCO-F EEFs were still able to import iron. Conversely, and as already reported, treatment of infected cells with 100 μΜ Deferoxamine (DFO), a high affinity chelator of Fe^3+^, drastically reduced the size of WT EEFs (90% decrease in size), such that they were smaller than ZIPCO-F in normal media (*P* = 3.6 × 10^−46^, Fig 5). Notably, DFO further decreased the size of ZIPCO-F EEFs by 80%, confirming that some import of iron takes place in the absence of ZIPCO (*P* = 1.1 × 10^−38,^ Fig 5). Finally, we confirmed that the effect of DFO was due to the chelation of iron by comparing EEF growth in media containing 100 μM DFO + 50 μg/ml FAC. In these conditions, the size of WT EEFs was comparable to growth in normal media or media supplemented with iron (FAC), showing that iron reverses the effect of DFO. Similarly, the size of ZIPCO EEFs was comparable to that of mutant EEFs grown in media supplemented by FAC, confirming that iron in FAC is in excess of DFO chelating capacity.

Taken together, these data show that iron is limiting for the growth of ZIPCO EEFs. The observation that mutant parasites have some growth potential in normal media, and their size increases with FAC suggests that these parasites are using another low affinity iron transporter to obtain iron. If so, they would be expected to be more sensitive to DFO treatment than WT-F EEFs. To test this, we compared the growth of WT-F and ZIPCO-F parasites in media with 10 μM DFO. Results show that in 10 μM DFO, WT-F EEFs are about 10 times bigger than in 100 μM DFO (60% of their size in normal media). On the contrary, ZIPCO-F parasites are as small in 10 μM DFO as in 100 μM DFO, indicating that they are indeed more sensitive to DFO than WT-F parasites.

### The ZIPCO phenotype is due to the lack of ZIPCO

RT-PCR analysis of ZIPCO-F liver-stage mRNAs detected a transcript corresponding to the 5′ part of the disrupted gene, but no transcript corresponding to the 3′ part downstream of the marker (Supplementary Fig S7). To exclude the possibility that the phenotype of the ZIPCO-F parasites might be due to expression of a 5′ ZIPCO truncate and some dominant negative effect, rather than the absence of full-length ZIPCO, we replaced the entire *ZIPCO* coding sequence by the selectable marker in the WT-F background by homologous recombination (Supplementary Fig S8A). A parasite clone was selected, named ZIPCO-ko, which was verified by PCR and Southern blot analysis of genomic DNA (Supplementary Fig S8B and C). As expected, the ZIPCO-ko phenotype recapitulated that of the ZIPCO-F and ZIPCO clones by displaying: normal growth during blood infection, normal mosquito infection capacity generating normal numbers of salivary gland sporozoites, and reduced sporozoite infectivity that can be seen from the 3 day delay in obtaining a parasitemia of 0.1%. (Supplementary Table S1 and Supplementary Fig S8D). Furthermore, ZIOCO-ko EEFs showed a 55% reduction in size at 48h postinfection (Supplementary Fig S8E). Therefore, the phenotype of all mutant clones is due to the lack of ZIPCO function.

## Discussion

We characterized here a *P. berghei* protein belonging to the ZIP family, called ZIPCO. The available expression data (see PlasmodDB) together with our analyses show that *ZIPCO* mRNA is detected in many *Plasmodium* parasite stages, including mixed blood stages, midgut and salivary gland sporozoites, and liver stages. However, to date neither ZIPCO in *P. berghei* nor its orthologs in *P. yoelli* or in *P. falciparum* have been detected in the published proteomic studies of different stages of the *Plasmodium* life cycle (PlasmoDB version 10.0). ZIPCO is specifically crucial for the development of the liver stage of the parasite during schizogony, and at 48 and 63 h, it appears to localize primarily at the liver-stage plasma membrane. We found no evidence that ZIPCO might be exported in the host cell cytoplasm or at the host cell surface.

Members of the ZIP family of proteins, which were first identified in *A. thaliana* and *S.cerevisae*, are found throughout the animal and plant kingdoms. ZIP family proteins are responsible for the transport of essential divalent cations, primarily iron and zinc, and thus play an important role in numerous cellular metabolic pathways (Eng *et al*, 1998; Guerinot, 2000). The largest pool of iron in mammals is associated with heme and is required for oxygen transport in haemoglobin, oxygen storage in myoglobin, and electron transport for cytochrome function in mitochondrial respiration. Iron is also essential for DNA replication, as a cofactor of ribonucleotide reductases (Sanvisens *et al*, 2011) and metalloproteins that contain an iron–sulphur (Fe-S) cluster (Wu & Brosh, 2012). In mammalian cells, ZIP proteins maintain the cytosolic concentration of metal ions by transporting them into the cell from the extracellular environment and possibly from intracellular compartments (Kambe *et al*, 2004; Liuzzi & Cousins, 2004; Eide, 2006). In protozoa, members of the ZIP family of proteins have been identified in the genomes of *Trypanosoma brucei* (Guerinot, 2000) and *Leishmania amazonensis* (Huynh *et al*, 2006). Recent studies have demonstrated that *L. amazonensis* acquires iron from the host macrophage via the conserved ZIP protein called *Leishmania* iron transporter 1 (LIT-1). LIT-1 is located at the parasite plasma membrane and is essential for parasite replication in macrophage phagolysosomes (Huynh *et al*, 2006; Huynh & Andrews, 2008; Jacques *et al*, 2010).

**Figure 6:**
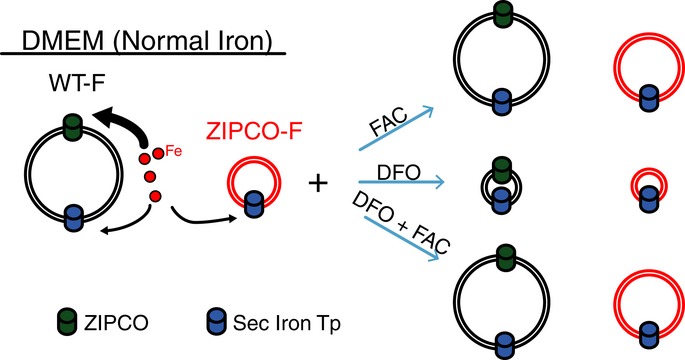
Proposed model of ZIPCO's role in iron homeostasis during *P. berghei* liver-stage development Based on our analyses of the sizes of WT-F and ZIPCO-F liver stages in normal media and media supplemented with or depleted of iron, together with the increased sensitivity of ZIPCO-F parasites to the iron chelator (DFO), we propose that WT-F liver stages (black circle) possess at least two iron transporters, ZIPCO shown as green cylinder and a second shown in blue. WT-F parasites grown in DMEM obtain sufficient amounts of iron for normal development. ZIPCO-F mutant parasites (shown in red) lack the iron transport activity of ZIPCO and use the second transporter to obtain some, but insufficient amounts, of iron for normal growth. Supplementing the media with ferric ammonium citrate (FAC) does not increase the growth of WT-F parasites since they obtain sufficient iron. On the contrary, extra iron in the media stimulates the growth of ZIPCO-F parasites. The presence of DFO, an iron chelator, removes iron and thereby blocks the growth of both WT-F and ZIPCO-F parasites. The effect of DFO is indeed due to the removal of iron since DFO + FAC results in normal growth of WT-F parasites and improved growth of ZIPCO-F mutants.

The lack of functional ZIPCO impaired liver-stage growth and viability and was associated with limited karyokinesis and resulted in a smaller number of hepatic merozoites. The phenotype of the ZIPCO mutant is similar to that of the wild type in hepatocytes depleted of iron (Stahel *et al*, 1988), including *in vivo* after hepcidin secretion (Portugal *et al*, 2011). Hepcidin, which is upregulated by increased levels of serum iron and by inflammatory cytokines such as IL-6, is induced by *Plasmodium* blood-stage infection and was indeed shown to inhibit concomitant development of superinfecting liver stages (Portugal *et al*, 2011). The ZIPCO mutant defect was reverted by iron supplementation, which further confirms that the phenotype of the mutant is due to iron deficiency. These results, together with the conserved ZIP signature, strongly suggest that ZIPCO is a transporter of iron that maintains iron homeostasis during liver-stage development of *Plasmodium* (see model proposed in Fig 6).

The *Plasmodium* genome contains several genes that code for iron or metal transporters other than ZIPCO. The liver stage itself displays some ZIPCO-independent iron-import capacity. Indeed, the complementation of the ZIPCO mutant defect by iron/FAC supplementation indicates iron can be imported in the liver stage in the absence of ZIPCO. Moreover, iron chelation further decreased the size of ZIPCO mutant liver stage and drastically decreased the growth of the wild-type liver stage to a size smaller than that of the ZIPCO mutant in normal conditions, clearly suggesting that liver stage express iron transport ability in addition to ZIPCO. The ZIPCO-encoding gene, which is conserved in *Plasmodium* species, has an equally conserved paralog in the *Plasmodium* genome, which might have such a low efficiency contribution to iron import by the liver stage. Interestingly, however, the gene encoding the ZIPCO paralog in *P. falciparum* (PF3D7_0609100) is expressed at low levels in erythrocytic schizonts but high levels in gametocytes and ookinetes, suggesting that it might play a role during sporogony in the oocyst, a nuclear division process that produces thousands of sporozoites from a single diploid zygote/multiploid ookinete. How intra-erythrocytic parasites import iron also remains unknown. The parasite intra-erythrocytic multiplication cycle imparts only a limited number of nuclear divisions, producing 10–30 merozoites from a single merozoite. Another lower-efficiency iron transporter, distinct from the ZIP family, might be involved in the blood stages, which might also be expressed in liver stages and responsible for the limited schizogony displayed by the ZIPCO mutant. Further work will tell how the various stages of *Plasmodium* acquire iron, and whether the ZIP containing proteins are only required in the stages that undergo extensive DNA replication, that is oocysts and liver stages.

Zinc supplementation had no effect on the size of WT liver stages but did increase the size of mutant EEFs, showing that the mutant phenotype is also due to zinc deficiency. Interestingly, zinc was shown elegantly by single-cell quantitative imaging to accumulate in parasite blood stages, maximally in mature parasites (schizonts), and to be important for the development of intra-erythrocytic parasite stages (Marvin *et al*, 2012). Zinc restriction by TPEN leads to blood-stage parasite death in a process that involves mitochondrial dysfunction, while zinc supplementation reverses this effect. Taken together, our results show that ZIPCO is required for the liver-stage parasite to obtain sufficient iron and zinc for its normal development and suggest, albeit indirectly, that ZIPCO is involved in the transport of these metal ions.

Iron has long been viewed as a potential target for intervention to fight against malaria. The antimalarial activity of iron chelators has now been demonstrated in animal models (Fritsch *et al*, 1985; Hershko *et al*, 1992), and these compounds have been developed for drug treatment in humans (for review see Weinberg & Moon, 2009). The recent discovery of hepcidin-induced liver-stage growth inhibition via iron deprivation has led to the idea of manipulating this interplay as an anti-liver-stage strategy (Prentice, 2011). In our quest to limit iron available to the pathogen, iron transporters in the parasite itself would appear as a direct and potent means to limit parasite growth. ZIPCO, and possible paralogs, might constitute good drug targets in our fight against the liver stage of the parasite, which remains to the most refractory to drug treatment.

## Materials and Methods

### Animals, parasites and cells

Three- to five-week-old female Swiss or C57Bl/6j mice and female Wistar rats were obtained from Janvier, France. All experiments using rodents were performed in accordance with the guidelines and regulations of the Pasteur Institute and were approved by the Ethical Committee for Animal Experimentation (registration number 2013-0093). *Anopheles stephensi*, strain Sda500, mosquitoes were reared at the Centre for Production and Infection of Anopheles (CEPIA) at the Pasteur Institute. The reference (wild-type, WT) *P. berghei* strains are NK65 and ANKA-GFP (obtained from MR4, MRA-867). In ANKA-GFP, GFP is expressed from the promoter *eef1*α throughout the life cycle (Janse *et al*, 2006a). The ANKA strain expressing RFP from the *eef1*α promoter (ANKA-L733) is described in Sturm *et al* (2009). *P. berghei* parasites were maintained by cycling between Swiss mice and mosquitoes, as described previously (Thiberge *et al*, 2007). The course of blood infections in mice was monitored on Giemsa-stained blood smears or by flow cytometry analysis. The number of sporozoites per salivary gland was counted on day 21–26 postinfection of mosquitoes. The human hepatoma cell line HepG2 was obtained from ATCC (HB-8065). Cells were maintained in Dulbecco's modified Eagle's medium (DMEM) + Glutamax (Gibco^**®**^) and 10% foetal calf serum (FCS) at 37°C and 5% CO_2_. The hybridoma producing the anti-circumsporozoite protein antibody was obtained from MR4, (MRA-100).

### Generation of ZIPCO and ZIPCO-F parasites

Plasmids for generating ZIPCO and ZIPCO-ko parasites were constructed by cloning 5′ and 3′ genomic sequences of *zipco* on either side of the hDHFR cassette in the pBC vector (Boisson *et al*, 2011) between *Apa*I and *Sma*I sites and *Not*I and *Asc*I sites, respectively (Supplementary Table S3 for primers used). ZIPCO recombinant parasites contain a deletion of 540 nucleotides that includes part of exon-2, intron-2, exon-3, intron-3 and part of exon-4 and are replaced by 1,601 nucleotides of the hDFHR cassette (see Fig 2A). This deletion corresponds to 84 amino acids of ZIPCO including the entire 4^th^ trans-membrane domain, the ZIP signature sequence and conserved histidine and glycine residues, and part of 5th trans-membrane domain. ZIPCO-ko recombinant parasites have a complete deletion of the predicted ORF (see Supplementary Fig S8A). Genetic manipulation of *P. berghei* was performed as previously described (Janse *et al*, 2006b; Lacroix *et al*, 2011). Briefly, NK65 and ANKA-GFP merozoites were electroporated with plasmid DNA digested with *Apa*I and *Asc*I. Recombinant parasites were selected by adding 0.07 mg/ml pyrimethamine (Sigma) to the drinking water of mice. Pyrimethamine- resistant parasites were cloned in mice by limiting dilution.

### Generation of ZIPCO-HA parasites

The entire *zipco* gene (1.4 kb) was cloned between *Apa*I and *Cla*I sites (for primers used see Supplementary Table S3), upstream of the HA-FLAG sequence in the pBC-HA-hDHFR plasmid described in Boisson *et al* (2011). The 3′ UTR of *zipco* (1 kb) was introduced between *Not*I and *Asc*I sites (see Supplementary Fig S5A). The resulting plasmid pBC-*zipco*-HA was sequenced, and the HA sequence was found to be in phase with the carboxy terminus of ZIPCO. The plasmid was digested with *Apa*I and *Asc*I and transfected into ANKA-GFP (WT-F) merozoites as described above. During the cloning procedure, the growth of ZIPCO-HA clones was comparable to WT-F as assessed by similar parasitemia (0.5–2%) on D8 postinfection.

### Generation of ZIPCO-ko parasites

For details on the generation of ZIPCO-ko parasites see Supplementary Fig S8.

### Genomic analysis of recombinant parasites

Southern blot analysis was performed on WT, WT-F, ZIPCO, ZIPCO-F, ZIPCO-ko and ZIPCO-HA recombinant parasites as previously described (Lacroix *et al*, 2011) using a Digoxygenin-labelled PCR-Probe and hybridization kit (Roche). Genomic DNA from the WT, WT-F, ZIPCO and ZIPCO-F blood-stage parasites were digested with *Bsm*I and *Hinc*II. Genomic DNA from WT-F and ZIPCO-HA blood-stage parasites were digested with *Bts*I and *Hpa*I (see Supplementary Fig S5B). Genomic DNA from WT-F and ZIPCO-ko were digested with *Pci*I and *Kpn*I. Membrane pre-hybridization, blocking, hybridization and detection were carried out according to manufacturer's instructions (Roche).

### Primers used in this study

Primers used for cloning, generating probes for Southern analysis, sequencing, RT-PCR of *zipco* transcripts and qPCR analysis are indicated in Supplementary Table S3.

### Quantitative RT-PCR analysis

Quantitative reverse transcription polymerase reaction was performed as described in Boisson *et al* (2011). Primers used: qPCR-F, qPCR-R, hsp70F and hsp70R.

### RT-PCR analysis of *zipco* transcripts

RT-PCR was performed on total RNA isolated from HepG2 cells 24 and 48 h postinfection with WT-F and ZIPCO-F sporozoites. Different primer combinations were used to detect full length or truncated transcripts of *zipco* (for details see primer list in Supplementary Table S3). Genomic DNA of WT-F and ZIPCO-F were used as positive controls for specific primer sets.

### *In vivo* infectivity of sporozoites

Freshly collected salivary gland sporozoites were injected intravenously or intradermaly into 3-week-old female C57Bl/6j mice (Janvier, France). Parasitemia was calculated daily from day 3 postinfection by flow cytometry or Geimsa staining of thin blood smears.

### *In vitro* development of EEFs

HepG2 cells were seeded onto 8-well collagen-treated Lab-Tek glass slides in DMEM + Glutamax + 10% FCS media supplemented with a cocktail of antibiotics (Penicillin 100 U/ml, streptomycin 100 μg/ml and neomycin 200 μg/ml). Freshly dissected sporozoites were added to the cells the next day (multiplicity of infection 0.5–1). Cultures were incubated at 37°C with 5% CO_2_ and 10% O_2_. Media was changed twice daily.

### *In vivo* development of EEFs

C57Bl/6j mice, 4 weeks old, were injected intravenously with 150,000 sporozoites and 24, 48 and 65 h postinfection mice were killed by cervical dislocation. The large liver lobe was removed, washed and images of developing EEFs were acquired using 50× Oil emersion objective on a Nataxix Inverted Objective Microscope. EEF size measurement was performed with ImageJ software (Schneider *et al*, 2012).

### Sporozoite gliding assay

The trail assay was performed as described earlier (Carey *et al*, 2013). Briefly, freshly dissected salivary gland sporozoites were incubated at 37°C, 5% CO_2_ and 10% O_2_ in DMEM containing 10% FCS for 45 min in poly-lysine coated 8-well IBID slide. Sporozoites were then fixed with 4% paraformaldehyde (PFA) for 10 min and washed with PBS and stained with anti-CS antibody (Yoshida *et al*, 1980) conjugated to Alexa-568 fluorophore.

### Cell traversal assay

The assay was performed as described in Giovannini *et al* (2011). Briefly, HepG2 cells (5 × 10^4^) were seeded in 8-well chamber slides in DMEM + Glutamax media containing 10% FCS and incubated at 37°C, 5% CO_2_ for 24 h. Freshly dissected day 21 salivary gland sporozoites were added to the cells in the presence of 2.0 mg/ml Rhodamin-dextran, incubated at 37°C for 1 h and then washed twice with PBS. Cells were detached with trypsin and fixed with 1% PFA in PBS supplemented with 10% FCS and analysed by flow cytometry for Rhodamin positive cells. HepG2 cells scratched with a sterile blade, in presence of Rhodamin-dextran, were used as a positive control for wounding and unscratched HepG2 cells incubated with Rhodamin-dextran used as a negative control. Equal numbers of WT-F and ZIPCO-F sporozoites were used per well (multiplicity of infection 0.5–1). All experiments were performed in triplicate except negative control.

### Immunofluorescence analysis of EEF size

For IFA of EEFs, HepG2 cells were infected with sporozoites in media supplemented with a cocktail of antibiotics as described above. After 2 h, cells were washed twice with PBS and media containing antibiotics were added. The medium was changed twice daily. Cells were fixed at 24, 48 and 65 h postinfection, with 4% PFA for 10 min, permeabilized with 0.05% Triton X-100 in PBS for 15 min and then washed with PBS. Samples were blocked over night at 4°C with 10% FBS in PBS. EEFs at 24 h were labelled with anti-UIS4 (kindly provided by Dr. S. Kappe) and 48 and 65 h EEFs with a chicken anti-EXP1 antibody (Sturm *et al*, 2009; kindly provided by Dr. V. Heussler). After several washes, samples were incubated with Alexa Fluor (568 or 647) conjugated secondary antibodies (Molecular probes).

### Immunolocalization of ZIPCO-HA

HepG2 cells were infected with WT-F or ZIPCO-HA sporozoites, and samples were fixed with 4% PFA at 48 h postinfection. After incubation with rat monoclonal anti-HA (Roche) and a rabbit anti *P. berghei* MSP1 polyclonal serum (de Koning-Ward *et al*, 2003) followed by Alexa Fluor 568 and 647 conjugated antibodies, samples were stained with DAPI and mounted in ProLong® Antifade kit (Molecular probes). 16-bit images were acquired at the Imaging Platform of the Pasteur Institute (PFID) on a laser scanning confocal microscope (LSM 700 Zeiss) with a 63× Plan-APOCHROMAT/oil objective (Zeiss). Images were processed using the ImageJ software (Schneider *et al*, 2012).

### *In vitro* development of hepatic merozoites

Fifty thousand HepG2 cells were plated per well (μ-slide 8 well, Ibidi) the day before infection with sporozoites. The cells were infected with 25,000 WT-RFP, WT-GFP or ZIPCO-F sporozoites per well. The slides were centrifuged for 5 min at 50 *g* at 20°C and incubated at 37°C, 5% CO_2_, 10% O_2_. Medium (DMEM + Glutamax Gibco^**®**^ + 10% FCS + 2% Penicillin–Streptomycin–Neomycin Solution, SIGMA) was changed at 24 h and 48 h postinfection. Supernatants were removed 66 and 90 h postinfection, filtered on a 5 μm filter (Minisart, Sartorius) and centrifuged for 3 min at 13,523 *g* at room temperature in 2 ml dolphin microcentrifuge tubes (Sorenson™). The pellet was resuspended in 100 μl PBS, and the number of GFP and RFP merozoites were counted in a cell count Kova slide on a Zeiss Axio Z1 microscope and 40× objective.

### Supplementation with Zinc or Iron and Iron deprivation with DFO

All chemicals were purchased from Sigma. HepG2 cells were infected with WT-F or ZIPCO-F sporozoites and 2 h postinfection fresh media containing different chemicals were added. The following concentrations were used: 20 μM ZnCl_2_; 10 or 100 μM Deferoxamine mesylate salt (DFO) and 50 μg/ml ferric ammonium citrate (FAC). Media was changed twice daily. Images of the EEFs were acquired 46 h postinfection with Zeiss Axio Z1 microscope using 50× objective. The sizes of GFP-expressing EEFs were measured on pictures using a custom ImageJ macro. Briefly, 12-bit images of the GFP-expressing EEFs were converted into 8-bit images and binarized using the ImageJ-implemented Intermodes thresholding method. When necessary, the ImageJ Watershed algorithm was applied to discriminate adjacent EEFs. The ImageJ Particle Analyzer was then used to determine the area of the binarized EEFs. Out-of-focus or inadequately segmented EEFs were excluded from the analysis.

### Statistical analyses

Quantitative RT-PCR data in Supplementary Fig S1 were analysed by Wilcox test. The number and sizes of EEFs in Fig 3 and Supplementary Fig S8E were analysed by independent two-tailed Student's *t*-tests with Graph Pad Prism (Version-6). The analysis of the effect of zinc and iron supplementation on EEF size shown in Fig 5, Supplementary Fig S6 and Supplementary Table S4 was conducted within treatments across replicates using a non-parametric test, followed by combination of *P*-values from independent tests of significance using the meta-analytical approach of Fisher (Fisher, 1932). Briefly, each treatment condition was compared to the control condition (DMEM) for each biological replicate independently, using the exact two-sided Wilcoxon rank sum test. Then, the *P*-values for each treatment were combined using Fisher's method. The threshold for significance was defined as *P* = 0.001. All computations were done using the R statistical software (version 2.14.1; R Development Core Team, 2011; http://www.r-project.org/) and the exact RankTests package (version 0.8-22). See also Supplementary Table S4.
