# Supplementary material for: ZIPCO, a putative metal ion transporter, is crucial for Plasmodium liver-stage development
Source: EMBO Mol Med. 2014 Sep 25;6(11):1387–97. doi: 10.15252/emmm.201403868 (PMC4237467; doi:10.15252/emmm.201403868)
Supplement: Supplementary file 1 [file emmm0006-1387-sd1.pdf]

ZIPCO, a putative iron transporter, is crucial for *Plasmodium* liver-stage development

Tejram Sahu, Bertrand Boisson, Céline Lacroix, Emmanuel Bischoff, Quentin Richier, Pauline Formaglio, Sabine Thiberge, Irina Dobrescu, Robert Ménard and Patricia Baldacci

Corresponding author: Patricia Baldacci, Institut Pasteur , submitted by Schuster

Review timeline:

Submission date:	16 January 2014
Editorial Decision:	12 February 2014
Revision received:	13 June 2014
Editorial Decision:	08 July 2014
Revision received:	23 July 2014
Accepted:	24 July 2014

Transaction Report:

(Note: With the exception of the correction of typographical or spelling errors that could be a source of ambiguity, letters and reports are not edited. The original formatting of letters and referee reports may not be reflected in this compilation.)

Editor: Roberto Buccione

1st Editorial Decision 12 February 2014

Thank you for the submission of your manuscript to EMBO Molecular Medicine. We have now heard back from the three Reviewers whom we asked to evaluate your manuscript.
You will see that while all three Reviewers are supportive of your work, they do express a number of important concerns, one of which fundamental and shared by all three, which prevent us from considering publication at this time. I will not dwell into much detail, as the evaluations are detailed and self-explanatory.

Reviewer 1 while appreciating your work, points to one main shortcoming, i.e. the evidence that ZIPCO is a bona fide iron transporter is not sufficient and that, as they stand, the findings might have alternative explanations. S/he suggests some possible approaches to address the issue.

Reviewer 2 has the exact same reservation and again similarly to Reviewer 1, suggests a possible strategy to solve the problem.

Reviewer 3 discusses a number of issues, including several important missing controls that are related to the parasitic aspect of your work and which require your action. Of note, this Reviewer, similarly to Reviewers 1 and 2, would like definitive evidence of the role of iron.

As you see, to establish the iron transporter function of ZIPCO with good confidence is necessary. It is up to you to achieve this according to the strategy you see appropriate and feasible.

Considered all the above, while publication of the paper cannot be considered at this stage, we would be pleased to consider a revised submission, with the understanding that the Reviewers'

concerns must be fully addressed, with additional experimental data where appropriate and that acceptance of the manuscript will entail a second round of review.

I look forward to seeing a revised form of your manuscript as soon as possible.

***** Reviewer's comments *****

Referee #1 (Remarks):

Sahu et al present evidence that ZIPCO is a Plasmodium gene that, when disrupted, causes partial but not total late liver stage arrest of parasites without affecting the blood- or mosquito stages. This is a nice series of experiments and the evidence is convincing.
However the evidence that ZIPCO is an iron transporter is less convincing. It is appreciated the authors employ the qualification 'putative' in the title of the manuscript but nevertheless in my view the impact of the paper would be clearer and greater if the iron-related nature of the growth defect of the ZIPCO deficient strain was further substantiated.
There are other knockouts (fatty acid synthase) that are also known to block development specifically during the late liver stage http://www.ncbi.nlm.nih.gov/pubmed/19068099 so that other metabolic defects may be responsible.
The evidence that ZIPCO is a liver-stage iron transporter depends significantly on figure 5 showing that increased FAC increases the EEF sizes of the mutant; but this is an in vitro experiment performed independently only twice. Furthermore it is a little surprising that iron only enhances the EEF growth of the ZIPCO mutant and not the wild-type, when previous work (eg Portugal et al Nat Med 2011) has shown that iron can enhance the in vitro and in vivo growth of wild-type liver-stages.

In my view the authors need to provide some extra substantiation to the idea that ZIPCO mutants lead to an inhibition of liver-stage infection because of a defect in iron accumulation. Two possibilities are: injection into mice of iron / use of iron-rich diets to test whether these enhance the in vivo growth of the mutant more than the wild-type; or testing whether wild-type ZIPCO can rescue growth in yeast mutants that lack iron importers (this would be a convincing finding).

Referee #2 (Comments on Novelty/Model System):

I think the significance/novelty of the work really stands on the review of an expert in malaria parasitism. My review can address the metal aspect of the work but not that broader issue.

Referee #2 (Remarks):

This manuscript identifies a ZIP family metal transporter as important for the infectivity of the malaria parasite Plasmodium. Overall the work is well done and clearly presented but the evidence that ZIPCO is indeed an iron transporter is weak.

Major comments:

The hypothesis that ZIPCO is an iron transporter, as opposed to other metals, is really based on the increase in EEF size in response to Fe treatment. More evidence is needed to support this conclusion. A simple way to further address this would be to express the protein in S cerevisiae mutants disrupted for zinc or iron uptake and test whether ZIPCO can suppress growth defects caused by those mutations.

The abstract refers to "deprivation" experiments indicating that iron is ZIPCO's substrate. I presume this is referring to the DFO experiments in Supporting Figure S6. This statement is not justified given that both WT and ZIPCO strains are sensitive to DFO. A DFO titration experiment should be done to show that ZIPCO is hypersensitive to DFO (relative to its corresponding untreated control) if this claim is to be made.

Minor comments:

Figure 2A and B. What is the source of the small fragment (~0.9 kb) that is specific to the ZIPCO alleles?

Figure 3A legend. When the authors say "... as shown in panel B.", do they mean panel D?

Supporting Table 1 title: "infectivity" is misspelled.

Supporting Figure S1. Using a stress-inducible gene (hsp70) seems like an odd choice of control mRNA. Is it known to not change during these various stages of infection?

Referee #3 (Comments on Novelty/Model System):

The medical impact is medium because the organism infects rodents not humans; however, the ZIPCO gene appears to be highly conserved and so there is a reasonable chance that the protein functions similarly in parasites that infect humans. Data using parasites that infect humans would be helpful.

Referee #3 (Remarks):

The manuscript by Sahu et al is an interesting and well written study describing a protein whose RNA message is upregulated in both sporozoites and exoerythrocytic stages of P. berghei, and which appears important for development of late-stage liver schizonts. While the identification of its importance to liver-stage parasites has been partly demonstrated (see major points below for additional controls required) the precise function of ZIPCO, or its mechanism of action, remain unknown. This comment is not intended to detract from the exciting results already obtained, or their novelty, but raises the question of whether mechanism or function is required for publication in EMBO Molecular Medicine.

To briefly summarize the manuscript: the authors show that ZIPCO is highly conserved in Plasmodium and that its mRNA is upregulated in P. berghei sporozoites and EEFs in vitro. The authors generated parasites lacking part, or all, of the ZIPCO gene, or where ZIPCO was HA-tagged at the coboxy terminus. Assessment of the mutants by light, immunofluorescence and intravital microscopy demonstrated a severe growth defect in parasites lacking ZIPCO and a delay in patency to blood-stage infection. This was elegantly confirmed by competition experiments using mCherry-expressing WT parasites as they developed into merozomes. ZIPCO mutant EEFs displayed poor karyokinesis but some merozoites were capable of infecting subsequent erythrocytes, indicating that the protein is important, but not essential, for development through the liver-stage. Addition of iron, in the form of ferric ammonium citrate (FAC), but not zinc, partially rescued the growth defect, implicating iron in the phenotype; however, full rescue was not obtained in the experiments presented. Overall this is a nice paper that adds new information to the literature but parts require clarification or additional controls. I have a few major points that require revision before recommending acceptance at EMBO Molecular Medicine. These changes are listed solely to further strengthen the paper towards removing any doubt that the phenotypes observed are completely attributable to ZIPCO.

Major points.
1. While the genotype of ZIPCO-F was confirmed by Southern blot, the authors have not demonstrated loss of ZIPCO gene expression. This should be shown. Either by Western blot of WT vs KO sporozoites (when expression is sufficiently high, see Figure S1), or at the very least, by Q-PCR.
2. In the absence of genetic complementation, the gold standard is to assess two independent KO clones to confirm the phenotypes observed are attributable only to the gene of interest. While both ZIPCO-F and ZIPCO-ko were assessed in the paper and gave similar results, the genotype of ZIPKO-ko was only confirmed by PCR. Southern analysis of the genotype and Q-PCR analysis of loss of gene expression is needed, in addition to ZIPCO-F, to validate the mutation in both clones. Alternatively, the authors claim to have made a line called "ZIPCO" (NK65 background) - where is the data for this line? If there is no experimental data, it should be removed, or the data included.

3. An important control for the IFAs is a Western blot demonstrating that HA antibodies recognize ZIPCO-HA specifically. As Western blotting of EEFs is technically challenging (anti-HA IP of infected monolayers might work), probing sporozoite lysates with anti-HA antibodies would be very informative and should be included.
4. The localization of ZIPCO is speculative and far from certain from the data presented. This is rather unhelpful. The authors should include a control antibody that labels the parasite plasma membrane, for example MSP1 and attempt to quantify the co-localization. Since ZIPCO is expressed late in liver schizonts, MSP1 should also be expressed. The alternative would be to perform immunoelectron microscopy on ZIPCO-HA parasites to localize the protein to the appropriate membrane(s).
5. In Figure 5, addition of an iron-chelating agent that prevents FAC rescue would be an important control to provide direct evidence of the role of iron.

Minor points
1. Figure S2 is busy - can the conserved ZIP domain residues (e.g. shown in Figure 1) be made clearer?
2. On pages 5 and 6, the conclusion is that "ZIPCO is not important" in the blood-stage. This interpretation is too strong and should be toned down to something like "not essential".
3. Figure 2D needs a legend included, as per 2C and 2E.
4. On page 7 it is stated: "The ZIPCO-HA clone infected mosquitoes as WT". This is unclear and took several re-reads. Should it say "... to the same degree as WT."?
5. Can the authors mention in the text whether any ZIPCO-HA could be seen before 48 hr post hepatocyte infection? The very high degree of mRNA expression in sporozoites (which should be confirmed by Western; see point above) implies the protein may be used early in hepatocyte infection. If this was not observed, it would be helpful to include this information. If it is unknown, it should be straightforward to assess.
6. It was not clear whether ZIPCO-HA was made in the WT or WT-F background. Perhaps it was the latter, which is why the IFAs show EXP1/GFP - please clarify this in the text.
7. In the discussion, more description of the potential role of ZIPCO in iron homeostasis is needed. Since ZIPCO-F partialy responds to FAC, it would suggest the protein is unlikely the sole main membrane channel; either a second channel may exist, or ZIPCO regulates the main channel. Further, the non-responsiveness to zinc treatment may indicate that ZIPCO is a zinc transporter. A model summarizing these possibilities would be most helpful.
8. Regarding point 7 above, on page 11 the discussion states: "These data strongly suggests that ZIPCO is the main transporter of iron....". This is not so, since the knockout does respond to FAC. If there were no channel there would be no response. The authors go on to tone this down in subsequent sentences, but the main sentence should also be toned down.
9. The addition of TPEN to intrahepatic WT parasites would quickly determine whether zinc is important for liver-stage development. This would add nicely towards the possibility that ZIPCO may be a zinc transporter, since the mutant did not respond to zinc.

1st Revision - authors' response 13 June 2014

<u>General response to Reviewers</u>

We thank the reviewers for their valuable input and largely agree with their criticisms. The shared view of the reviewers is that the importance of ZIPCO in liver stage development is quite clear, however whether ZIPCO acts by transporting iron and/or other metals was not conclusively shown.

Toward a better characterization of ZIPCO function, two reviewers suggested trying to perform rescue experiments of yeast mutants lacking iron importers, as this would be a convincing finding. We totally agree and indeed we had made several attempts, in collaboration with G. Breard, C. Saveneau and L. Decourty in the Unit of Genetics of Macromolecular Interactions at the Institut Pasteur, before completing the first draft of the paper. First, the *Plasmodium* ZIPCO sequence was cloned in pCM190-HA. Several yeast strains, the WT (BY4741) and several metal ion transporter mutants were transformed with the plasmid and colonies were obtained that grew normally, indicating that the plasmodium sequence was not toxic to cells. However, when protein extracts were analysed by commassie staining or by western blot with an anti HA-HRP antibody we could

not detect a band at 35kDa, the expected size for ZIPCO (see panels A and B in Figure below). In the absence of detectable ZIPCO expression we did not attempt the complementation experiments. Next, we tried to obtain ZIPCO expression in yeast using a codon-optimized sequence in the pCM-190 and pCM-190HA. Again, we did not obtain correct expression of ZIPCO in yeast. Despite a correct sequence and expression being repressed by doxycycline as expected, western blot analysis of crude protein extracts using an anti HA-HRP antibody revealed many bands but no clear band at 35kDa corresponding to the expected size of ZIPCO-HA (see panel C in Figure below). These multiple bands are probably due to the formation of protein aggregates.

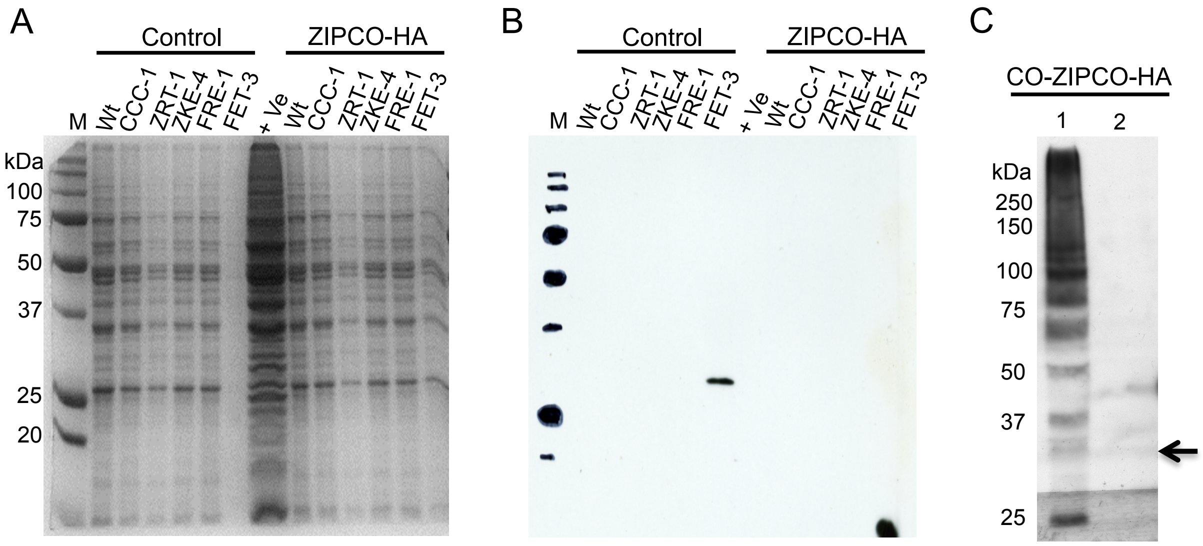

Figure 1: Western blot analysis of crude yeast protein extracts with anti-HA-HRP antibody.

Panel A: Commassie staining of crude protein extracts of Wt and mutant yeast colonies transformed with vector alone (Control) or vector with P. berghei ZIPCO coding sequence. No extra band at 35kDa was observed in ZIPCO-HA clones.

Panel B: Western blot of the same protein extracts as in A, probed with an anti HA-HRP antibody. Again no specific band was observed in ZIPCO-HA transformants compared to controls.

Panel C: Western blot of WT yeast strain transformed with codon optimized sequence of ZIPCO-HA grown in the absence (lane 1) or presence (lane2) of doxycycline. The arrow indicates weak band of size expected for ZIPCO-HA.

Given the presence of the weak band at about 35kDa, we tried nevertheless to complement several yeast mutants: FRE1, deficient in ferric reductase that is required for uptake of ferric ion; FET3 deficient in high affinity Fe(II) transport; ATX2, deficient in manganese transport and ZRT1, deficient for a high affinity zinc transporter with the ZIPCO-HA plasmid, hoping there might be sufficient ZIPCO protein to complement the mutants. We also tested the ZIPCO codon optimized construct without the HA tag. Disappointingly, no complementation of the yeast mutants was obtained.

Therefore our yeast rescue experiments were inconclusive, possibly due to incorrect expression of ZIPCO. We cannot exclude that correct production of the protein might require additional parasite factors, such as the expression of a chaperone, or other conditions, for example using another expression vector.

Nonetheless, we have further tested the hypothesis of ZIPCO acting as an iron and/or zinc transporter by performing more experiments based on iron and zinc addition or depletion to further pinpoint ZIPCO activity. Experiments are summarized in the new Figure 5).

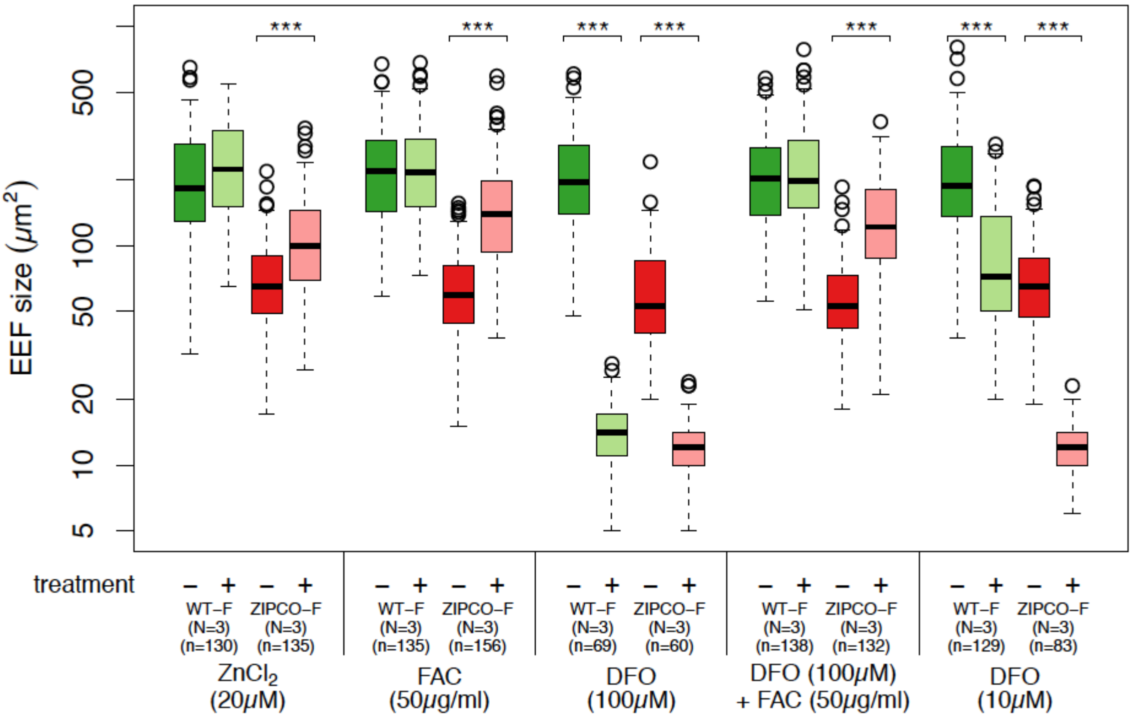

All controls requested by reviewers were performed, and we further tested the role of zinc in the mutant phenotype. Results clearly indicate that iron increases the size of mutant EEFs, although iron is still imported to low levels in the mutant. They also suggest a role of zinc in the mutant phenotype. This is a conceptual change compared to the first draft of the manuscript. These data strongly suggest that ZIPCO imports iron and possibly zinc in the liver stage. The Expanded View Table E4 shown below lists the experiments performed in this study and shows statistical analysis.

Table E4: **statistical analysis of the effect of Zinc and Iron on EEF size.** Analysis was conducted within treatments across replicates using a non- parametric test, followed by combination of P values from independent tests of significance using the meta- analytical approach of Fisher (Fisher, 1932). Briefly, each treatment condition was compared to the control condition (DMEM) for each biological replicates independently, using the exact two sided Wilcoxon rank sum test. Then, the P values for each treatment were combined using Fisher's method. The threshold for significance was defined as p= 0.001. All computations were done using the R statistical software (version 2.14.1; R Development Core Team, 2011) and the exactRankTests package (version 0.8- 22; Hothorn and Hornik, 2011).

				EEF	EEF size					Fisher's combined probability		
**experiment**	**condition**	**parasite**	**replicate**	**#**	**median**	**mean**	**sd**	**Z-statistic**	**p-value**	**chi2**	**df**	**p-value**
ZnCl2_20	DMEM	WT-F	rep1	52	222	232.3	110.1	-0.64	0.52			
ZnCl2_20	ZnCl2_20	WT-F	rep1	51	234	244.8	106.4					
ZnCl2_20	DMEM	WT-F	rep2	38	171	209.2	114.6	-1.5	0.15	10.61	6	0.1
ZnCl2_20	ZnCl2_20	WT-F	rep2	45	208	241.1	117.2					
ZnCl2_20	DMEM	WT-F	rep3	37	162	207.3	134.4	-1.8	0.065			
ZnCl2_20	ZnCl2_20	WT-F	rep3	34	236	250.8	116.1					
ZnCl2_20	DMEM	ZIPCO-F	rep1	54	69	80.5	38.6	-1.8	0.08			
ZnCl2_20	ZnCl2_20	ZIPCO-F	rep1	53	82	94.1	49.3					
ZnCl2_20	DMEM	ZIPCO-F	rep2	44	56	58.6	25.5	-5.1	9.1e-08	63.93	6	7.1e-12
ZnCl2_20	ZnCl2_20	ZIPCO-F	rep2	46	99.5	121	70.7					
ZnCl2_20	DMEM	ZIPCO-F	rep3	31	75	79.1	29	-4.6	1.8e-06			
ZnCl2_20	ZnCl2_20	ZIPCO-F	rep3	36	128	129.4	47.9					
FAC50	DMEM	WT-F	rep1	46	205.5	230.9	113.9	1.8	0.078			
FAC50	FAC50	WT-F	rep1	48	169.5	187.3	77.1					
FAC50	DMEM	WT-F	rep2	44	203	243.5	136.1	-1.3	0.19	10.03	6	0.12
FAC50	FAC50	WT-F	rep2	43	236	271.7	129.4					
FAC50	DMEM	WT-F	rep3	40	231.5	236.3	112.7	-0.77	0.44			
FAC50	FAC50	WT-F	rep3	44	232	265.9	135.1					
FAC50	DMEM	ZIPCO-F	rep1	45	50	59.7	27.3	-6.5	3.9e-12			
FAC50	FAC50	ZIPCO-F	rep1	68	109	124.4	67.4					
FAC50	DMEM	ZIPCO-F	rep2	49	61	63.6	24.9	-7.3	1.1e-16	179.9	6	3.6e-36
FAC50	FAC50	ZIPCO-F	rep2	42	177	178.9	76.3					
FAC50	DMEM	ZIPCO-F	rep3	38	71	76.1	32.3	-6.4	2,00E-12			
FAC50	FAC50	ZIPCO-F	rep3	46	173	193.7	113.2					
DFO100	DMEM	WT-F	rep1	44	186	217.5	126.2	6.4	5.1e-17			
DFO100	DFO100	WT-F	rep1	20	12.5	12.3	4.9					
DFO100	DMEM	WT-F	rep2	33	207	225	109.4	6.6	5.1e-17	226.9	6	3.6e-46
DFO100	DFO100	WT-F	rep2	26	17	17.6	3.9					
DFO100	DMEM	WT-F	rep3	40	206.5	211.2	104.6	6.6	2.1e-17			
DFO100	DFO100	WT-F	rep3	23	13	13.3	3.4					
DFO100	DMEM	ZIPCO-F	rep1	36	43.5	46.6	18.9	5.7	9.6e-14			
DFO100	DFO100	ZIPCO-F	rep1	16	9.5	9.3	2.7					
DFO100	DMEM	ZIPCO-F	rep2	32	53	58.5	21.9	6.1	9.4e-15	191.8	6	1.1e-38
DFO100	DFO100	ZIPCO-F	rep2	21	12	12.5	3.5					
DFO100	DMEM	ZIPCO-F	rep3	33	91	91	44.5					
DFO100	DFO100	ZIPCO-F	rep3	23	13	13.9	4.3	6.3	2.5e-15			
DFO100FAC50	DMEM	WT-F	rep1	49	181	197.3	90.8	-0.6	0.55			
DFO100FAC50	DFO100_FAC50	WT-F	rep1	52	170	219.8	126.5					
DFO100FAC50	DMEM	WT-F	rep2	44	188	227	125.5	-1.4	0.17	6.946	6	0.33
DFO100FAC50	DFO100_FAC50	WT-F	rep2	48	216.5	259	146.8					
DFO100FAC50	DMEM	WT-F	rep3	41	225	238.8	109.8	0.97	0.33			
DFO100FAC50	DFO100_FAC50	WT-F	rep3	38	198.5	220.4	122.1					
DFO100FAC50	DMEM	ZIPCO-F	rep1	46	47.5	53.2	19.9	-6	1.9e-10			
DFO100FAC50	DFO100_FAC50	ZIPCO-F	rep1	47	92	112.7	60					
DFO100FAC50	DMEM	ZIPCO-F	rep2	40	50	51.7	16.8	-7.4	6.5e-18	155.2	6	6E-31
DFO100FAC50	DFO100_FAC50	ZIPCO-F	rep2	44	132.5	156.9	77.6					
DFO100FAC50	DMEM	ZIPCO-F	rep3	34	74	81.5	36.1	-5	1.6e-07			
DFO100FAC50	DFO100_FAC50	ZIPCO-F	rep3	41	142	151.6	66					
DFO10	DMEM	WT-F	rep1	42	162	218	147.2	6.2	2.2e-11			
DFO10	DFO10	WT-F	rep1	52	70	76.8	43.8					
DFO10	DMEM	WT-F	rep2	42	230	254	142.4	4.1	2.8e-05	126.5	6	7E-25
DFO10	DFO10	WT-F	rep2	44	139	144.3	68.1					
DFO10	DMEM	WT-F	rep3	34	179.5	192.1	80.7	6.3	5.5e-13			
DFO10	DFO10	WT-F	rep3	33	52	62.7	31.2					
DFO10	DMEM	ZIPCO-F	rep1	43	60	70.6	37	7.3	3,00E-21			
DFO10	DFO10	ZIPCO-F	rep1	31	11	11.2	3					
DFO10	DMEM	ZIPCO-F	rep2	41	63	63.8	26.6	7.1	3.4e-19	247.8	6	1.2e-50
DFO10	DFO10	ZIPCO-F	rep2	30	14	14.1	3.8					
DFO10	DMEM	ZIPCO-F	rep3	33	66	74.5	33.1	6.2	1.5e-15			
DFO10	DFO10	ZIPCO-F	rep3	22	11	11	2.4					

In conclusion, we think that our data, although lacking the definitive proof via yeast rescue, make a compelling case that ZIPCO imports both iron and possibly zinc in the *Plasmodium* liver stage, and plays a crucial role in parasite growth in the liver and merozoite production.

A new paragraph entitled "*ZIPCO* mutant deficiency is reversed by iron and zinc", page 8, in the revised version presents these improved and new data. The relevant part of the discussion, has also been changed, page 11. All changes are highlighted in yellow.

We have also changed a number of other parts of the text in keeping with reviewers' requests (also highlighted in yellow in the new draft). The title has been changed to integrate the possible role of zinc in the ZIPCO phenotype, which now conservatively presents ZIPCO as a putative 'metal ion' transporter, instead of 'iron' transporter.

Referee #1:

Sahu et al present evidence that ZIPCO is a Plasmodium gene that, when disrupted, causes partial but not total late liver stage arrest of parasites without affecting the blood- or mosquito stages. This is a nice series of experiments and the evidence is convincing. However the evidence that ZIPCO is an iron transporter is less convincing. It is appreciated the authors employ the qualification 'putative' in the title of the manuscript but nevertheless in my view the impact of the paper would be clearer and greater if the iron-related nature of the growth defect of the ZIPCO deficient strain was further substantiated. There are other knockouts (fatty acid synthase) that are also known to block development specifically during the late liver stage http://www.ncbi.nlm.nih.gov/pubmed/19068099 so that other metabolic defects may be responsible. The evidence that ZIPCO is a liver-stage iron transporter depends significantly on figure 5 showing that increased FAC increases the EEF sizes of the mutant; but this is an in vitro experiment performed independently only twice.

R: We have repeated that experiment and added three new experimental replicates (see new figure 5 and Expanded View Table E4). FAC significantly increases the size of ZIPCO mutant EEFs, as previously described. These data indicate that iron complements ZIPCO liver-stage growth deficiency and thus strongly suggest that ZIPCO transports iron.

Furthermore it is a little surprising that iron only enhances the EEF growth of the ZIPCO mutant and not the wild-type, when previous work (eg Portugal et al Nat Med 2011) has shown that iron can enhance the in vitro and in vivo growth of wild-type liver-stages.

R: This is correct, and indeed we did not highlight this discrepancy in the previous draft. We repeated these experiments, and new data (see figure above, new Fig 5) confirm our original data.

It is difficult to elaborate on the reasons for the difference between Portugal et al and our results. We note that Portugal's experiments used 100 mg/ml FAC, Huh7 cells and RPMI, while ours used 50 mg/ml FAC, HepG2 cells and DMEM.
One potentially important difference is the cell line used. Portugal et al used Huh-7 cells, while we used the reference HepG2 cell line. A paper (Vecchi C, Montosi G, and Pietrangelo A. 2010. Huh-7: A Human "Hemochromatotic" Cell Line, Hepatology 51, 654) reveals that Huh-7 cells carry a mutation in HFE (a major histocompatibility class I–like protein), which is similar to that associated with human hemochromatosis and impairs proper HFE traffic and interaction with the transferrin receptor. Huh-7 cells display lower messenger RNA levels of hepcidin compared to HepG2 cells, which carry a wild-type HFE. This might cause inappropriate iron sensing by Huh-7 hepatocytes.

Perhaps equally important is the fact that Portugal et al measured EEF size at 36 h, while we scored EEF size at 46 h, when ZIPCO mutant phenotype was most obvious. It is thus possible that WT EEFs in Portugal experiments grow faster in excess iron (difference noted at 36 h), but do not reach a bigger size at the end of EEF growth (>46 h).

We feel that our results obtained at 46 h are solid and that adding the above discursive part – even shortened - as to the possible reasons for the discrepancy with Portugal's data would cut the flow of the text, in the result or discussion parts.

In my view the authors need to provide some extra substantiation to the idea that ZIPCO mutants lead to an inhibition of liver-stage infection because of a defect in iron accumulation. Two possibilities are: injection into mice of iron / use of iron-rich diets to test whether these enhance the in vivo growth of the mutant more than the wild-type;

R: This could indeed be a way to test the hypothesis. However, imaging EEFs in vivo is not trivial (can only detect a few EEFs per animal) and to obtain enough images for valid statistical analysis would be very time and mice consuming. Furthermore, such experiments would also require tricky controls to determine if the iron rich or poor diet indeed reduces the amount of iron available in hepatocytes. For these reasons we have concentrated on the in vitro system, by confirming and expanding previous data.

... or testing whether wild-type ZIPCO can rescue growth in yeast mutants that lack iron importers (this would be a convincing finding).

R: As discussed above, we did try this approach but could not express ZIPCO in yeast nor obtain complementation of the mutants we tested.

Referee #2
(Comments on Novelty/Model System):
I think the significance/novelty of the work really stands on the review of an expert in malaria parasitism. My review can address the metal aspect of the work but not that broader issue.

Referee #2 (Remarks):
This manuscript identifies a ZIP family metal transporter as important for the infectivity of the malaria parasite Plasmodium. Overall the work is well done and clearly presented but the evidence that ZIPCO is indeed an iron transporter is weak.

Major comments:
The hypothesis that ZIPCO is an iron transporter, as opposed to other metals, is really based on the increase in EEF size in response to Fe treatment. More evidence is needed to support this conclusion. A simple way to further address this would be to express the protein in S cerevisiae mutants disrupted for zinc or iron uptake and test whether ZIPCO can suppress growth defects caused by those mutations.

R: We agree that complementation of yeast mutants would have been the most elegant demonstration but we could not obtain such evidence as discussed above.

The abstract refers to "deprivation" experiments indicating that iron is ZIPCO's substrate. I presume this is referring to the DFO experiments in Supporting Figure S6. This statement is not justified given that both WT and ZIPCO strains are sensitive to DFO. A DFO titration experiment should be done to show that ZIPCO is hypersensitive to DFO (relative to its corresponding untreated control) if this claim is to be made.

R: This is correct. We presented that both WT and ZIPCO were sensitive to 100 mM DFO, which is indeed not sufficient to say that iron is the substrate of ZIPCO. However, this statement was not based solely on the DFO experiment but, more importantly, on the supplementation with FAC. Nonetheless the reviewer is right to request a DFO titration experiment. We have performed this experiment, which show that at 10 mM DFO, ZIPCO-F EEFs are very small like in DFO 100 mM. In contrast, WT-F EEFs are only about half their normal size in DFO 10 mM, still significantly larger (10 times) than in DFO 100 mM (see new figure 5, and below). We therefore conclude that ZIPCO mutants are more sensitive to DFO than the WT.

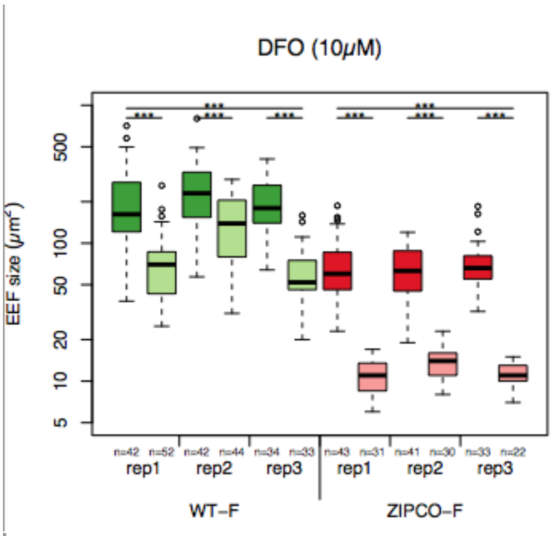

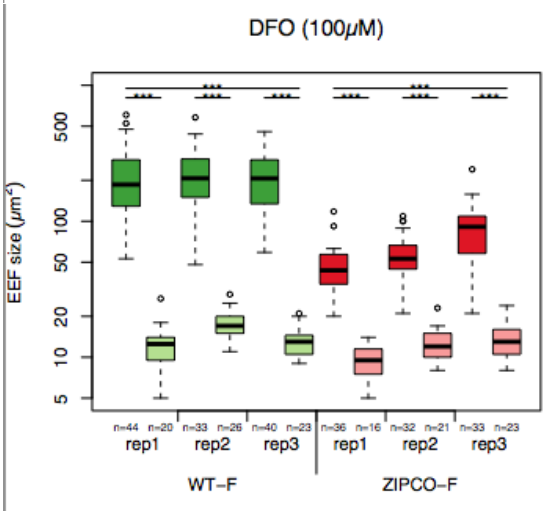

We have also performed experiments with 100 mM DFO + 50 mg/ml FAC (see new figure 5). Results show that the effect of DFO is indeed due to chelation of iron, since FAC reverses the inhibitory effect of DFO.

Minor comments:
Figure 2A and B. What is the source of the small fragment (~0.9 kb) that is specific to the ZIPCO alleles?

R: An extra band is present in the Southern presented Figure 2A. This band (0.9 kb) is due to the presence of another *Hinc*II site, which we omitted to show in the figure. The probe used detects this restriction fragment in both WT-F and ZIPCO-F parasites.

We have corrected this oversight and the figure now shows the second *Hinc*II site.

Figure 3A legend. When the authors say "... as shown in panel B.", do they mean panel D?

R: Again the reviewer is correct, this was a mistake, which is now corrected.

Supporting Table 1 title: "infectivity" is misspelled.

R: This is corrected

Supporting Figure S1. Using a stress-inducible gene (hsp70) seems like an odd choice of control mRNA. Is it known to not change during these various stages of infection?

R: There is no ideal control for q-pcr for the different parasite stages during the life cycle. Over the years, we have tried primers for other mRNAs and found hsp70 to be the most reliable (Rosinski-Chupin I, et al., BMC Genomics. 2007 *Serial Analysis of Gene Expression in Plasmodium berghei salivary gland sporozoites.)* We have used this reference gene in previous work for quantifying the expression of about a hundred genes expressed in sporozoite and liver stages, as described previously in Ishino et al. 2009 Cell Microbiol and Boisson et al. 2011 Mol Microbiol. More recently, another group has quantified the expression of hsp70 genes throughout the life cycle (Hlics, M. et al. 2013 Plos one: *Expression profiling of Plasmodium berghei HSP70 genes for generation of bright red fluorescent parasites*). Their data show that the gene coding for hsp70/3, used for normalisation in our q-pcr experiments, is stably expressed throughout the life cycle (blood stage merozoites, gametocytes, ookinetes, sporozoites, 24h and 48h liver stages). Altogether, we think that hsp70 is an appropriate reference gene and not such an odd choice.

Referee #3
(Comments on Novelty/Model System):
The medical impact is medium because the organism infects rodents not humans; however, the ZIPCO gene appears to be highly conserved and so there is a reasonable chance that the protein functions similarly in parasites that infect humans. Data using parasites that infect humans would be helpful.

R: Most molecular studies on *Plasmodium* liver stages, especially those dealing with parasite protein function, are performed using rodent-infecting *Plasmodium* species like *P. berghei* or *P. yoelii*. Handling *P. falciparum* mosquito and pre-erythrocytic stages is heavily constrained - *P. falciparum* sporozoites only invade primary human hepatocytes, where infection levels remain low. In addition, because ZIPCO displays 59% identity and 76% similarity between human- and rodent-infecting species, we feel it is highly likely that what we report here for *P. berghei* holds true, at least qualitatively, for *P. falciparum*.

(Remarks):
The manuscript by Sahu et al is an interesting and well written study describing a protein whose RNA message is upregulated in both sporozoites and exoerythrocytic stages of P. berghei, and which appears important for development of late-stage liver schizonts. While the identification of its importance to liver-stage parasites has been partly demonstrated (see major points below for additional controls required) the precise function of ZIPCO, or its mechanism of action, remain unknown. This comment is not intended to detract from the exciting results already obtained, or their novelty, but raises the question of whether mechanism or function is required for publication in EMBO Molecular Medicine.

R: We believe we have added new experiments/arguments that strengthen our conclusion that ZIPCO acts as an iron and possibly a zinc transporter, and that transport of both metals is likely to play a role in the mutant phenotype.

To briefly summarize the manuscript: the authors show that ZIPCO is highly conserved in Plasmodium and that its mRNA is upregulated in P. berghei sporozoites and EEFs in vitro. The authors generated parasites lacking part, or all, of the ZIPCO gene, or where ZIPCO was HA-tagged at the coboxy terminus. Assessment of the mutants by light, immunofluorescence and intravital microscopy demonstrated a severe growth defect in parasites lacking ZIPCO and a delay in patency to blood-stage infection. This was elegantly confirmed by competition experiments using mCherry-expressing WT parasites as they developed into merozomes. ZIPCO mutant EEFs displayed poor karyokinesis but some merozoites were capable of infecting subsequent erythrocytes, indicating that the protein is important, but not essential, for development through the liver-stage. Addition of iron, in the form of ferric ammonium citrate (FAC), but not zinc, partially rescued the growth defect, implicating iron in the phenotype; however, full rescue was not obtained in the experiments presented.

Overall this is a nice paper that adds new information to the literature but parts require clarification or additional controls. I have a few major points that require revision before recommending acceptance at EMBO Molecular Medicine. These changes are listed solely to further strengthen the paper towards removing any doubt that the phenotypes observed are completely attributable to ZIPCO.

Major points.
1. While the genotype of ZIPCO-F was confirmed by Southern blot, the authors have not demonstrated loss of ZIPCO gene expression. This should be shown. Either by Western blot of WT vs KO sporozoites (when expression is sufficiently high, see Figure S1), or at the very least, by Q-PCR.

R: Western blot on sporozoites is currently not feasible since we do not have good antibodies directed against ZIPCO, which is the reason why we constructed the HA tagged version of ZIPCO to localize the protein.

Additionally, although ZIPCO mRNA is indeed present in sporozoites, we have not been able to detect ZIPCO-HA (which is functional since it replaces endogenous ZIPCO) in sporozoites by IFA or by western blot.

The result of the western blot on ZIPCO-HA sporozoites is now shown in Fig E5, panel C.

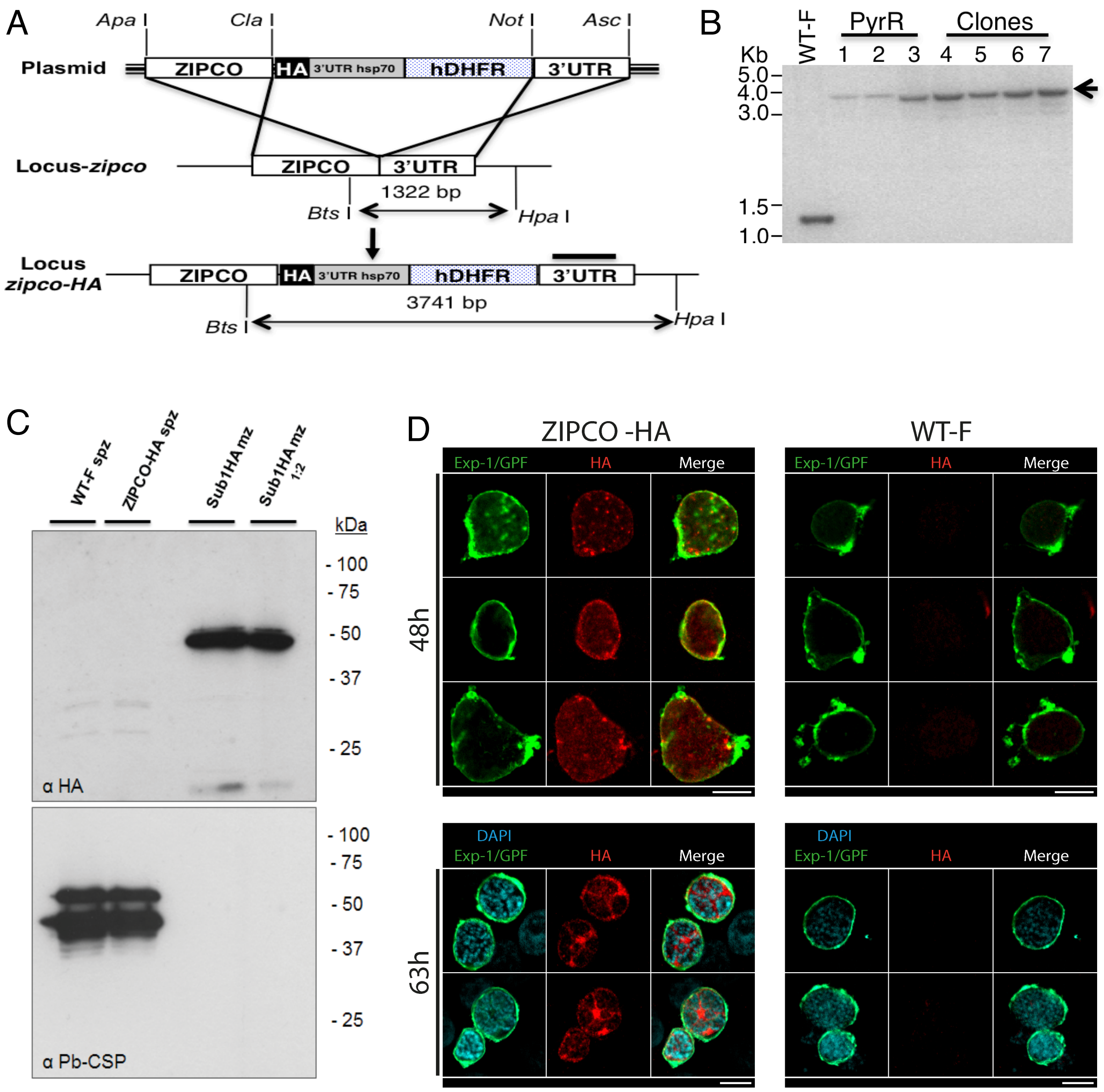

Thus, although *zipco* mRNA is detected in sporozoites we cannot detect the protein. We only detect the expression of ZIPCO-HA in late liver stages as shown in new figure 4 and above in panel D.

We showed by RT-PCR that there is a partial ZIPCO transcript corresponding to the region upstream of the hDHFR cassette, but no detectable transcript of the region downstream the cassette, which is predicted to mediate ion transport. However, although the partial transcript is not expected to have transport activity, it could potentially give rise to a truncated protein causing the mutant phenotype by a 'dominant negative' effect. This is why we generated the complete deletion of ZIPCO in zipco-ko parasites, which cannot express ZIPCO mRNA. Overall, we can conclude that ZIPCO activity is absent in our mutants.

2. In the absence of genetic complementation, the gold standard is to assess two independent KO clones to confirm the phenotypes observed are attributable only to the gene of interest. While both ZIPCO-F and ZIPCO-ko were assessed in the paper and gave similar results, the genotype of ZIPKO-ko was only confirmed by PCR. Southern analysis of the genotype and Q-PCR analysis of loss of gene expression is needed, in addition to ZIPCO-F, to validate the mutation in both clones.

R: A Southern blot analysis of the ZIPCO-KO mutant has been performed as requested, which is shown in figure E8. The blot confirms the correct genotype of this mutant and the lack of ZIPCO coding sequence.

Alternatively, the authors claim to have made a line called "ZIPCO" (NK65 background) - where is the data for this line? If there is no experimental data, it should be removed, or the data included.

R: The experimental data concerning the ZIPCO mutant in the NK65 background was included in the initial manuscript, but not indeed highlighted adequately. With this mutant, we have studied parasite infectivity to mosquitoes (Table E1), sporozoite infectivity to mice (Table E2) and the size of EEFs by IFA (Fig E4).

This is now phrased as follows, page 6, in the section “ZIPCO is important for EEF development” at end of first paragraph:

A similar delay was observed with ZIPCO sporozoites after IV injection (Table E2).

And in second paragraph:

This decrease in size was also observed in the EEFs of the recombinant clone ZIPCO (Fig E4A).

*3. An important control for the IFAs is a Western blot demonstrating that HA antibodies recognize ZIPCO-HA specifically. As Western blotting of EEFs is technically challenging (anti-HA IP of infected monolayers might work), probing sporozoite lysates with anti-HA antibodies would be very infor*mative and should be included.

R: We did this experiment, and we did not detect the ZIPCO-HA fusion in western blots of sporozoites using anti-HA antibodies, which we already used to label an NPT1 fusion (Boisson et al, 2011, Mol Microbiol) and were specific. These results are now shown in Fig E5.

4. The localization of ZIPCO is speculative and far from certain from the data presented. This is rather unhelpful. The authors should include a control antibody that labels the parasite plasma membrane, for example MSP1 and attempt to quantify the co-localization. Since ZIPCO is expressed late in liver schizonts, MSP1 should also be expressed.

R: We agree that previous images were not optimal.

We have performed new IFAs with anti-HA and MSP1 antibodies, images were acquired on a confocal microscope, and results are far better (new Figure 4, below). The use of anti HA and anti MSP antibodies indicates that, within the limits of the technique, ZIPCO and MSP1 at least partially co-localise. These data strongly suggest that ZIPCO associates with the parasite plasma membrane, like MSP1, especially in view of images of the mature schizont stage in panel C.

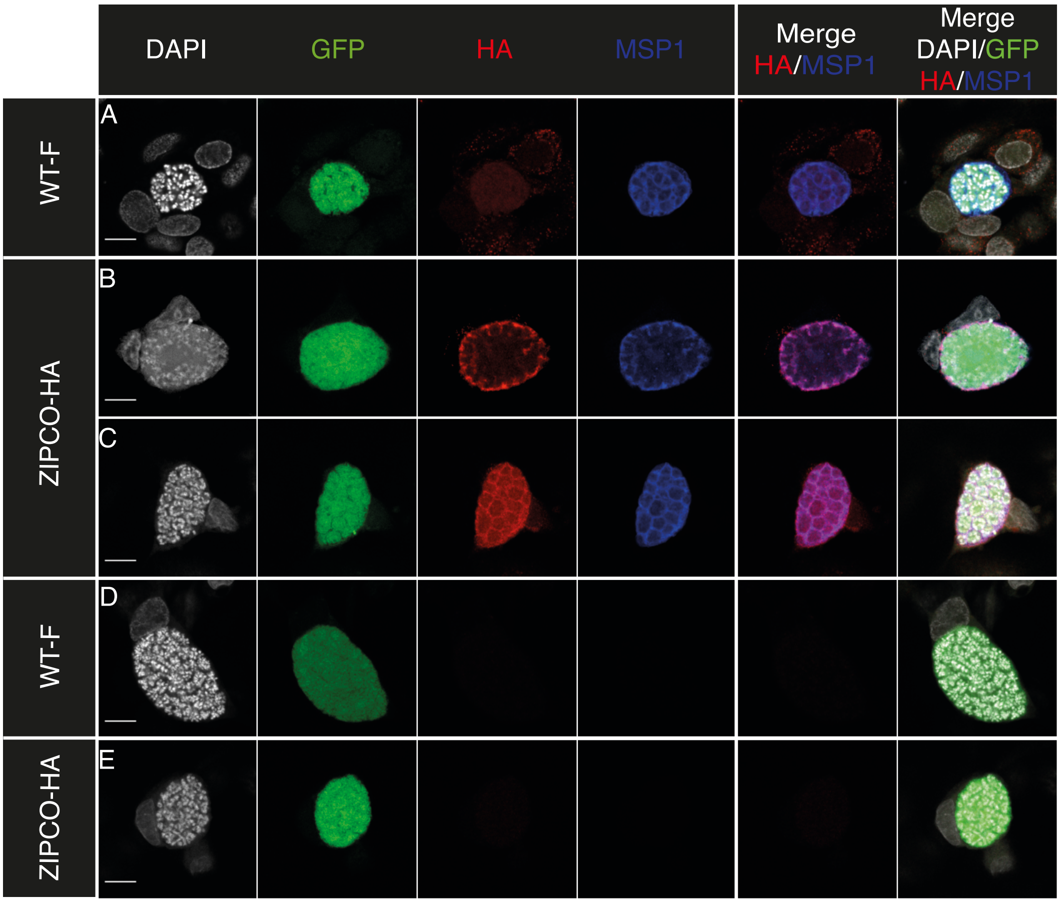

The text now reads:

To assess the expression of ZIPCO-HA in sporozoites, we performed a western blot using anti-HA monoclonal antibodies. As shown in Fig E5C, we were unable to detect a specific band at the expected 35kDa in ZIPCO-HA sporozoites.

To investigate ZIPCO-HA production by and localisation in liver stages, ZIPCO-HA infected HepG2 cells were stained using a monoclonal anti-HA antibody. A specific signal was detected in EEFs at 48 and 63 hpi (Fig 4 and Fig E5, panel D). At 48 hpi, a specific signal was detected, as a punctuated labelling within and at the periphery of the developing parasite (Fig 4, panel B). Double labelling with MSP-1, a parasite protein located at the plasma membrane, showed regions of overlap with the HA signal (Fig 4, panel B merged image). In more advanced EEFs at the cytomere stage (Graewe et al, 2011) the HA labelling was detected around groups of nuclei and again there was overlap with the MSP1 signal (Fig 4, panel C). These data strongly suggest that ZIPCO-HA is mainly located at the parasite plasma membrane.

The alternative would be to perform immunoelectron microscopy on ZIPCO-HA parasites to localize the protein to the appropriate membrane(s).

R: We have not performed immuno-TEM, since we do not have good anti-ZIPCO antibodies and although the anti HA antibody works well for western blot and IFA experiments, our previous experience using it in immunoTEM has been inconclusive. Indeed, we tried to use to this antibody

© EMBO

to localize another putative transport protein NPT1 in gametocytes (Boisson et al., 2011). We observed very few gold particles in both WT and NPT1-HA parasites.

5. In Figure 5, addition of an iron-chelating agent that prevents FAC rescue would be an important control to provide direct evidence of the role of iron.

R: We have performed these experiments and the results are shown in the new figure 5 and Table E4. Our data show that the use of 100 mM DFO + 50 mg/ml FAC equals the effect of 50 mg/ml FAC alone on EEF size, which shows that inhibition by DFO is indeed through iron chelation. The text now reads:

Notably, DFO further decreased the size of ZIPCO-F EEFs by 80%, confirming that some import of iron takes place in the absence of ZIPCO. Finally, we confirmed that the effect of DFO was due to the chelation of iron by comparing EEF growth in media containing 100 mM DFO + 50 mg/ml FAC. In these conditions, the size of WT EEFs was comparable to growth in normal media or media supplemented with iron (FAC), showing that iron reverses the effect of DFO. Similarly, the size of ZIPCO EEFs was comparable to that of mutant EEFs grown in media supplemented by FAC, confirming that iron in FAC is in excess of DFO chelating capacity.

Minor points
1. Figure S2 is busy - can the conserved ZIP domain residues (e.g. shown in Figure 1) be made clearer?

R: We have composed a new figure S2, which is reduced in size and just shows the region in and around the ZIP domain.

2. On pages 5 and 6, the conclusion is that "ZIPCO is not important" in the blood-stage. This interpretation is too strong and should be toned down to something like "not essential".

R: This was changed accordingly. Nonetheless, we feel that when a KO has no detectable phenotype in a process (here multiplication in RBC), it is legitimate to say that the product is not important for that process, which otherwise would imply the unlikely case of a 100% efficient adaptation/compensation mechanism. This is particularly true when the KO has a drastic phenotype in another process (here multiplication in hepatocytes).

3. Figure 2D needs a legend included, as per 2C and 2E.

R: This was an oversight, which is now corrected.

4. On page 7 it is stated: "The ZIPCO-HA clone infected mosquitoes as WT". This is unclear and took several re-reads. Should it say "... to the same degree as WT."?

R: This was modified accordingly.

5. Can the authors mention in the text whether any ZIPCO-HA could be seen before 48 hr post hepatocyte infection? The very high degree of mRNA expression in sporozoites (which should be confirmed by Western; see point above) implies the protein may be used early in hepatocyte infection. If this was not observed, it would be helpful to include this information. If it is unknown, it should be straightforward to assess.

R: We performed western blot analysis of sporozoite extracts using HA monoclonal antibodies. It appears that sporozoites do not express, or at very low levels, ZIPCO (see new Fig E5, panel C). The protein is only detected by IFA in late liver stages, where it is expressed at high levels (new Fig 4). These data suggest that while mRNA is present in sporozoites, the protein is only produced in liver stages, in agreement with the phenotype of mutants. However, it is nonetheless likely that

ZIPCO is produced early during liver stage development, as shown by the early/rapid growth delay of ZIPCO mutant.

This has been rephrased in the new text, which reads page 7-8:
To assess the expression of ZIPCO-HA in sporozoites, we performed a western blot using anti-HA monoclonal antibodies. As shown in Fig E5C, we were unable to detect a specific band at the expected 35kDa in ZIPCO-HA sporozoites.

To investigate ZIPCO-HA production by and localisation in liver stages, ZIPCO-HA infected HepG2 cells were stained using a monoclonal anti-HA antibody. A specific signal was detected in EEFs at 48 and 63 hpi (Fig 4 and Fig E5, panel D). At 48 hpi, a specific signal was detected, as a punctuated labelling within and at the periphery of the developing parasite (Fig 4, panel B). Double labelling with MSP-1, a parasite protein located at the plasma membrane, showed regions of overlap with the HA signal (Fig 4, panel B merged image). In more advanced EEFs at the cytomere stage (Graewe et al, 2011) the HA labelling was detected around groups of nuclei and again there was overlap with the MSP1 signal (Fig 4, panel C). These data strongly suggest that ZIPCO-HA is mainly located at the parasite plasma membrane.

6. It was not clear whether ZIPCO-HA was made in the WT or WT-F background. Perhaps it was the latter, which is why the IFAs show EXP1/GFP - please clarify this in the text.

R: This is correct: the ZIPCO-HA fusion was engineered in the WT-F background, as indicated in the methods section. For clarity we have now written this in the results section P7:

To address ZIPCO protein expression and subcellular localization, we generated by allelic exchange in the WT-F background the recombinant ZIPCO-HA parasite in which a HA tag was fused in frame to the carboxy terminus of ZIPCO (Fig E5A and B).

7. In the discussion, more description of the potential role of ZIPCO in iron homeostasis is needed. Since ZIPCO-F partialy responds to FAC, it would suggest the protein is unlikely the sole main membrane channel; either a second channel may exist, or ZIPCO regulates the main channel. Further, the non-responsiveness to zinc treatment may indicate that ZIPCO is a zinc transporter. A model summarizing these possibilities would be most helpful.

R: We indeed think that ZIPCO is not the only iron transporter, since DFO further decreases the size of ZIPCO mutant EEFs. This is discussed and now shown in a new Figure 6, which focuses on ZIPCO and iron transport, shown below.

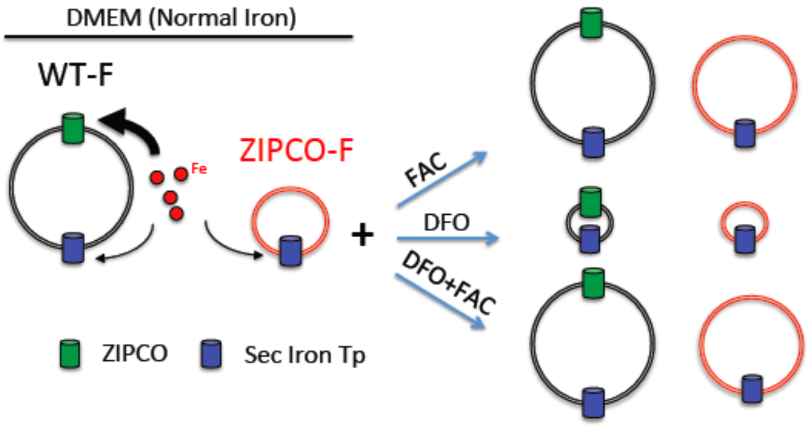

8. Regarding point 7 above, on page 11 the discussion states: "These data strongly suggests that ZIPCO is the main transporter of iron....". This is not so, since the knockout does respond to FAC. If there were no channel there would be no response. The authors go on to tone this down in subsequent sentences, but the main sentence should also be toned down.

R: Our data, including those newly obtained, clearly suggest that ZIPCO transports iron, and the phenotype of the ZIPCO mutant is complemented by FAC to a great extent. However, another iron transporter, which we presume to be of lower affinity, seems to exist in liver stages since DFO further decreases the size of ZIPCO mutant EEFs.

The text p11 in discussion now reads:

These data strongly suggest that ZIPCO is a transporter of iron that maintains iron homeostasis during liver-stage development of *Plasmodium.*

9. The addition of TPEN to intrahepatic WT parasites would quickly determine whether zinc is important for liver-stage development. This would add nicely towards the possibility that ZIPCO may be a zinc transporter, since the mutant did not respond to zinc.

R: The data from the new experiments with Zinc supplementation show that the ZIPCO-F mutant responds to Zinc suggesting that ZIPCO may be transporting zinc as well. We have done and repeated TPEN experiments many times. They yielded desperately inconsistent results at 2 mM, which is probably not enough to completely remove zinc, while higher concentrations of TPEN (5 mM) kill host cells. We have not been able to perform meaningful experiments with TPEN, which is therefore not mentioned.

This was added in the first paragraph of *ZIPCO* mutant deficiency is reversed by iron and zinc, which now reads:

To determine whether ZIPCO might be transporting zinc and/or iron, WT-F and ZIPCO-F EEFs were first grown in media supplemented with 20 mM zinc chloride ($ZnCl_2$). This treatment had no significant effect on the size of WT EEFs, but significantly increased the size of mutant EEFs at 46 hpi (Fig 5). This suggested that the mutant growth defect was partly due to zinc deficiency. To further test the role of zinc in EEF growth, we allowed EEFs to grow in the presence of TPEN, a zinc chelator. However, at 2 mM concentration, TPEN yielded inconsistent results, while higher concentrations of TPEN were toxic for host cells (not shown).

2nd Editorial Decision 08 July 2014

Thank you for the submission of your revised manuscript to EMBO Molecular Medicine. We have now received the enclosed reports from the Reviewers that were asked to re-assess it. As you will see the Reviewers are now globally supportive although there are a few remaining issues.

Briefly, Reviewer 2 asks for a few remaining corrections while Reviewer 3 would like you to tone down some passages in the manuscript for instance where you take for granted that ZIPCO is a metal ion transporter; please amend as suggested. When you submit your revised manuscript, please make sure you upload an additional manuscript file highlighting where these changes have been made.

Please also consider the following final Editorial amendments/requests:

1) The numbering on your expanded view figures is unclear. Please make sure you include the figure number in each expanded view figure as indicated in the legends.

2) Please indicate the actual P value for each test (not merely 'significant' or 'P < 0.05') for figures 5, E1 and E6.

***** Reviewer's comments *****

Referee #1 (Remarks):

The authors have adequately addressed the criticisms, and have toned down their interpretation. The findings are interesting, should be published and exposed to the community of malaria researchers.

Referee #2 (Remarks):

The authors have addressed all of my previous criticisms. At this stage, I have only minor revisions to recommend.

1) Abstract- I suggest changing "Iron/zinc complementation..." to "Iron/zinc supplementation..."

2) Page 3, paragraph 1. I believe that the authors are mistaken about hepcidin reducing hepatocyte iron. Hepcidin decreases plasma iron by, in part, increasing hepatocyte storage.

3) The legend to Figure 2B should be updated to also mention the symbol indicating the 949 bp fragment.

Referee #3 (Comments on Novelty/Model System):

Thank you for the opportunity to see the significantly revised manuscript.
I have now had a chance to go through all of the old, plus new, data and have read the new manuscript.
I am quite satisfied that the authors have addressed most of my concerns, in that they have included:

1. A Western blot on HA-tagged sporozoites was performed and no protein expression could be detected.
While this does not confirm the specificity of the antibody in their microscopy images, it does add new data on the expression profile of ZIPCO.

2. Evidence that the second knockout genotype is correct by Southern blot and qPCR data showing that ZIPCO mutants no longer express the wild-type transcript.

3. The inclusion of confocal microscopy with MSP1 co-localization, which really adds confidence to the claim that ZIPCO localizes at the parasite membrane.

4. The addition of DFO + FAC to confirm that the rescue observed by FAC is indeed the result of iron. I also particularly liked the 10 uM DFO experiment showing that ZIPCO mutants are more sensitive than WT.

5. The inclusion of a Model in Figure 6. However, the language is too strong and needs to be softened (see also below paragraph). For example, the figure title probably could read "Proposed model of ZIPCO's putative role in iron homeostasis...."

However, at present, all the evidence indicates that ZIPCO is needed to utilize iron and zinc, and its function as a metal ion transporter is but one possibility. Perhaps ZIPCO is a factor that regulates the transporter(s).
While the protein contains 6 transmembrane domains, plus a signal peptide, and shares significant homology to other membrane transporters, there is no biochemical evidence for this, or even that it is membrane-bound.

I do not think this should preclude publication (this is some very nice work of broad interest); however, all of the language throughout the paper requires toning down. For example, in the abstract, it should be said that "Iron/zinc complementation and depletion experiments indicate that ZIPCO is required for parasite utilization of iron and possibly zinc, consistent with ZIPCO's predicted function as a metal ion transporter" or something to that effect. And it also cannot be concluded that parasites must have two transporters. Following careful re-wording, I would feel that the paper warrants acceptance at EMBO Molecular Medicine.

2nd Revision - authors' response 23 July 2014

Our replies to the reviewers' comments are:

Referee #1 (Remarks): The authors have adequately addressed the criticisms, and have toned down their interpretation. The findings are interesting, should be published and exposed to the community of malaria researchers.

Referee #2 (Remarks): The authors have addressed all of my previous criticisms.
At this stage, I have only minor revisions to recommend.

1) Abstract- I suggest changing "Iron/zinc complementation..." to "Iron/zinc supplementation..."

Response: we have changed the text of the abstract accordingly. It now reads:

"Iron/zinc supplementation and depletion experiments suggest that ZIPCO is required for parasite utilization of iron, and possibly zinc, consistent with its predicted function as a metal transporter"

2) Page 3, paragraph 1. I believe that the authors are mistaken about hepcidin reducing hepatocyte iron. Hepcidin decreases plasma iron by, in part, increasing hepatocyte storage.

Response: The reviewer is correct. The sentence was not sufficiently precise. It has been changed and now reads:

« Induced upon infection or inflammation, hepcidin depletes iron from the plasma by binding to ferroportin thereby decreasing absorption from the intestine, recycling from macrophages and release of iron stored in hepatocytes »

3) The legend to Figure 2B should be updated to also mention the symbol indicating the 949 bp fragment.

Response: this has been done.

Referee #3 (Comments on Novelty/Model System): Thank you for the opportunity to see the significantly revised manuscript. I have now had a chance to go through all of the old, plus new, data and have read the new manuscript. I am quite satisfied that the authors have addressed most of my concerns, in that they have included:
1. A Western blot on HA-tagged sporozoites was performed and no protein expression could be detected. While this does not confirm the specificity of the antibody in their microscopy images, it does add new data on the expression profile of ZIPCO.
2. Evidence that the second knockout genotype is correct by Southern blot and qPCR data showing that ZIPCO mutants no longer express the wild-type transcript.
3. The inclusion of confocal microscopy with MSP1 co-localization, which really adds confidence to the claim that ZIPCO localizes at the parasite membrane.
4. The addition of DFO + FAC to confirm that the rescue observed by FAC is indeed the result of iron. I also particularly liked the 10 uM DFO experiment showing that ZIPCO mutants are more sensitive than WT.

5. The inclusion of a Model in Figure 6. However, the language is too strong and needs to be softened (see also below paragraph). For example, the figure title probably could read "Proposed model of ZIPCO's putative role in iron homeostasis...."

Response: The figure title has been changed as requested.

However, at present, all the evidence indicates that ZIPCO is needed to utilize iron and zinc, and its function as a metal ion transporter is but one possibility. Perhaps ZIPCO is a factor that regulates the transporter(s). While the protein contains 6 transmembrane domains, plus a signal peptide, and shares significant homology to other membrane transporters, there is no biochemical evidence for this, or even that it is membrane-bound. I do not think this should preclude publication (this is some very nice work of broad interest); however, all of the language throughout the paper requires toning down.

Response: Factually the reviewer is correct in saying that we have not demonstrated the iron or zinc transport activity of ZIPCO. However, considering the phenotype we report, the results of the depletion and supplementation experiments, together with the conserved ZIP signature and the annotation of the protein as a putative metal ion transporter, makes it extremely likely that ZIPCO is a transporter. Nevertheless we have tried to be as conservative as possible by writing « suggests » etc, and made some modifications to « tone down » the text (see below).

For example, in the abstract, it should be said that "Iron/zinc complementation and depletion experiments indicate that ZIPCO is required for parasite utilization of iron and possibly zinc, consistent with ZIPCO's predicted function as a metal ion transporter" or something to that effect.

Response: The text in the abstract has been modified as requested and now reads:

"Iron/zinc supplementation and depletion experiments suggest that ZIPCO is required for parasite utilization of iron, and possibly zinc, consistent with its predicted function as a metal transporter."

And it also cannot be concluded that parasites must have two transporters.

We consider that the data (the difference between the liver stage sizes of mutant vs WT + iron deprivation) suggest such a conclusion.

Following careful re-wording, I would feel that the paper warrants acceptance at EMBO Molecular Medicine.

Response: The discussion, Title and legend Figure 6 have been modified and now read:

Page 10-11. "These results, together with the conserved ZIP signature, strongly suggest that ZIPCO is a transporter of iron that maintains iron homeostasis during liver-stage development of *Plasmodium* (see model proposed in Fig 6)."

Page 11. "Zinc supplementation had no effect on the size of WT liver stages but did increase the size of mutant EEFs, showing that the mutant phenotype is also due to zinc deficiency."

P11. "Moreover, iron chelation further decreased the size of ZIPCO mutant liver stages and drastically decreased the growth of the wild-type liver stage to a size smaller than that of the ZIPCO mutant in normal conditions, suggesting that liver stages express iron transport ability in addition to ZIPCO."

Page 11-12. "Taken together, our results show that ZIPCO is required for the liver stage parasite to obtain sufficient iron and zinc for its normal development and suggest, albeit indirectly, that ZIPCO is involved in the transport of these metal ions."

Page 24. Title and Figure legend of Figure 6 have been modified and now read:
Figure 6. Proposed model of ZIPCO's role in iron homeostasis during *P. berghei* Liver Stage development.
Based on our analyses of the sizes of WT-F and ZIPCO-F liver stages in normal media and media supplemented with or depleted of iron, together with the increased sensitivity of ZIPCO-F parasites to the iron chelator (DFO), we propose that WT-F liver stages (black circle) possess at least two iron transporters, ZIPCO shown as green cylinder and a second shown in blue. WT-F parasites grown in DMEM obtain sufficient amounts of iron for normal development. ZIPCO-F mutant parasites (shown in red) lack the iron transport activity of ZIPCO and use the second transporter to obtain some, but insufficient amounts, of iron for normal growth. Supplementing the media with ferric ammonium citrate (FAC) does not increase the growth of WT-F parasites since they obtain sufficient iron. On the contrary, extra iron in the media stimulates the growth of ZIPCO-F parasites. The presence of DFO, an iron chelator, removes iron and thereby blocks the growth of both WT-F and ZIPCO-F parasites. The effect of DFO is indeed due to the removal of iron since DFO + FAC results in normal growth of WT-F parasites and improved growth of ZIPCO-F mutants.

Please also consider the following final Editorial amendments/requests:
1) The numbering on your expanded view figures is unclear. Please make sure you include the figure number in each expanded view figure as indicated in the legends.

The expanded view figures have been modified accordingly.

2) Please indicate the actual P value for each test (not merely 'significant' or 'P < 0.05') for figures 5, E1 and E6

We have added the significant p values to Fig 5 and added them in main text ; an addition has been made to the figure legend "Significant p values are indicated; refer to Table E4 for complete statistical analysis".
The p value for Figure E1 has been added to the legend and also put in main text.
We have not put the values on E6 because it makes it unreadable. We have added to the figure legend "The complete statistical analysis with p values is shown in Table E4".

3) Please provide the short list of bullet points that summarise the key NEW findings.

The bullet points are provided in separate file

4) We are now encouraging the publication of source data, particularly for electrophoretic gels and blots, with the aim of making primary data more accessible and transparent to the reader. Would you be willing to provide a PDF file per figure that contains the original, un-cropped and unprocessed scans of all or at least the key gels used in the manuscript? The PDF files should be labelled with the appropriate figure/panel number, and should have molecular weight markers; further annotation may be useful but is not essential. The PDF files will be published online with the article as supplementary "Source Data" files. If you have any questions regarding this just contact me.

We will upload the source data pdf files for :
Figure 2, Panel-B
Figure E5, Panel-B
Figure E5, Panel-C
Figure E7
Figure E8, Panel-B
Figure E8, Panel-C

© EMBO
