# Supplementary material for: ZIPCO, a putative metal ion transporter, is crucial for Plasmodium liver-stage development
Source: EMBO Mol Med. 2014 Sep 25;6(11):1387–97. doi: 10.15252/emmm.201403868 (PMC4237467; doi:10.15252/emmm.201403868)
Supplement: Supplementary file 2 [file emmm0006-1387-sd2.pdf]

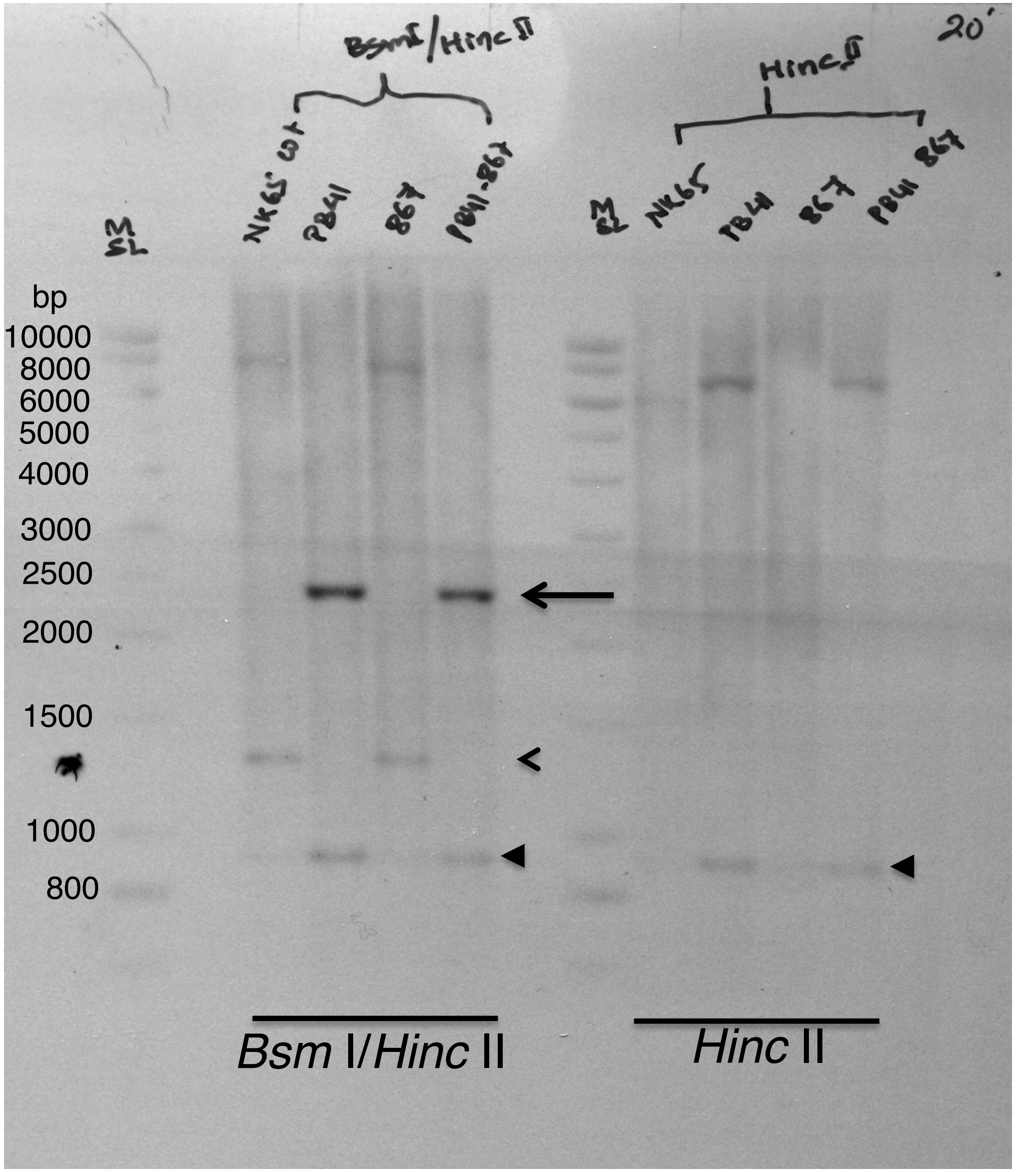

MSL: Mol Wt Marker (Smart ladder)
NK65 wt: WT
PB41: ZIPCO
867: WT-F
PB41-867: ZIPCO-F

Left Panel shows digestion with *Bsm* I and *Hinc* II

Right Panel shows digestion with *Hinc* II only

Recombinant band shown by arrow

WT band shown by open arrow-head

Hinc II band shown by solid arrow-heads

Figure 2, Panel-B: Analysis of *zipco* locus in WT and recombinant parasites.
