# Supplementary figures and images for: ZIPCO, a putative metal ion transporter, is crucial for Plasmodium liver-stage development
Source: EMBO Mol Med. 2014 Sep 25;6(11):1387–97. doi: 10.15252/emmm.201403868 (PMC4237467; doi:10.15252/emmm.201403868)

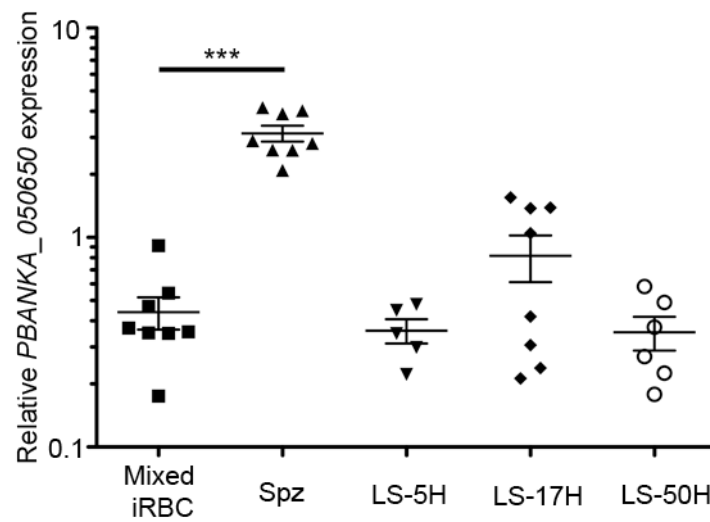

**Figure S1. Expression of *PBANKA\_050650* mRNA during the life cycle**

Supplement: Supplementary file 3 [file emmm0006-1387-sd3.pdf]

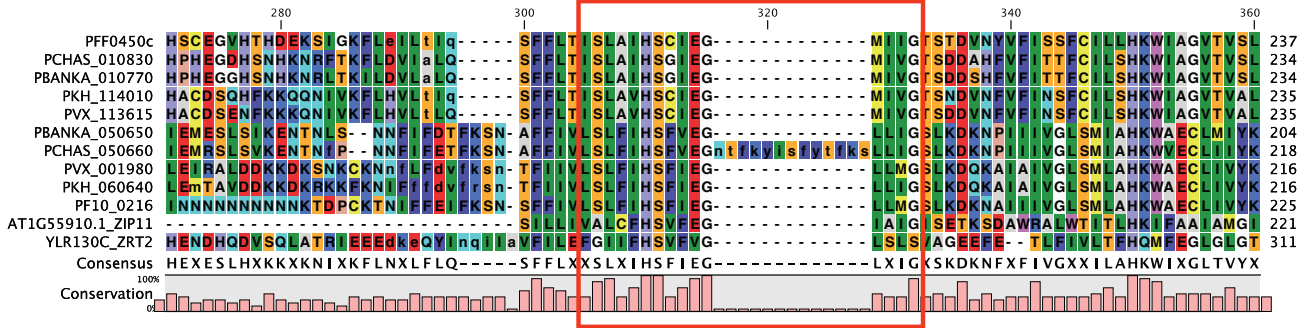

Figure S2. PBANKA\_050650 contains a conserved ZIP domain

Supplement: Supplementary file 4 [file emmm0006-1387-sd4.pdf]

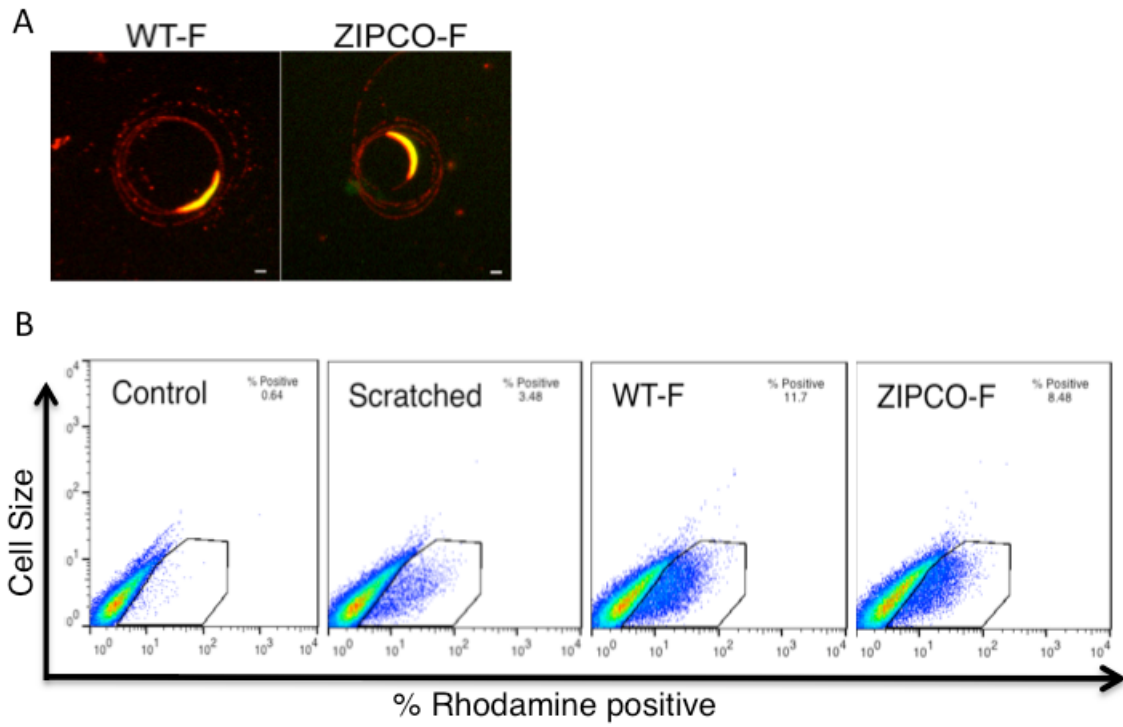

**Figure S3. ZIPCO-F sporozoites have normal gliding motility and cell traversal activity**

Supplement: Supplementary file 5 [file emmm0006-1387-sd5.pdf]

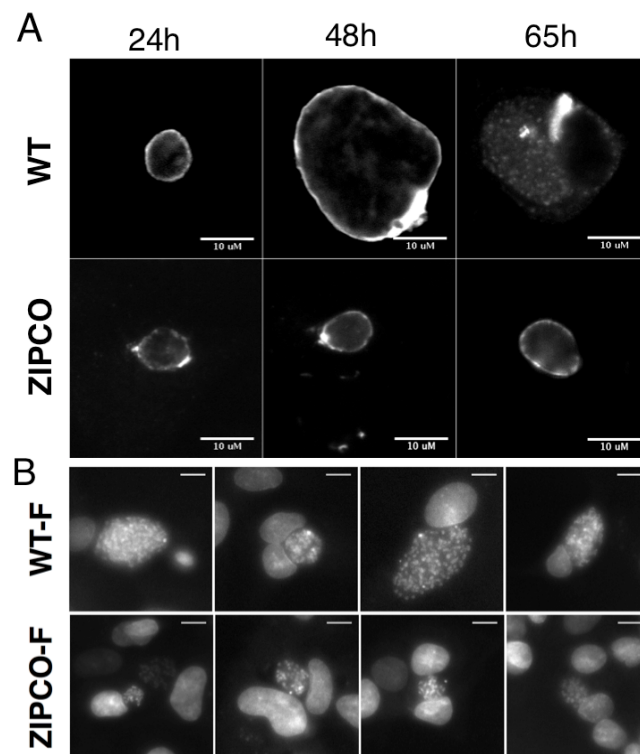

**Figure S4. ZIPCO parasites are defective in liver stage development**

Supplement: Supplementary file 6 [file emmm0006-1387-sd6.pdf]

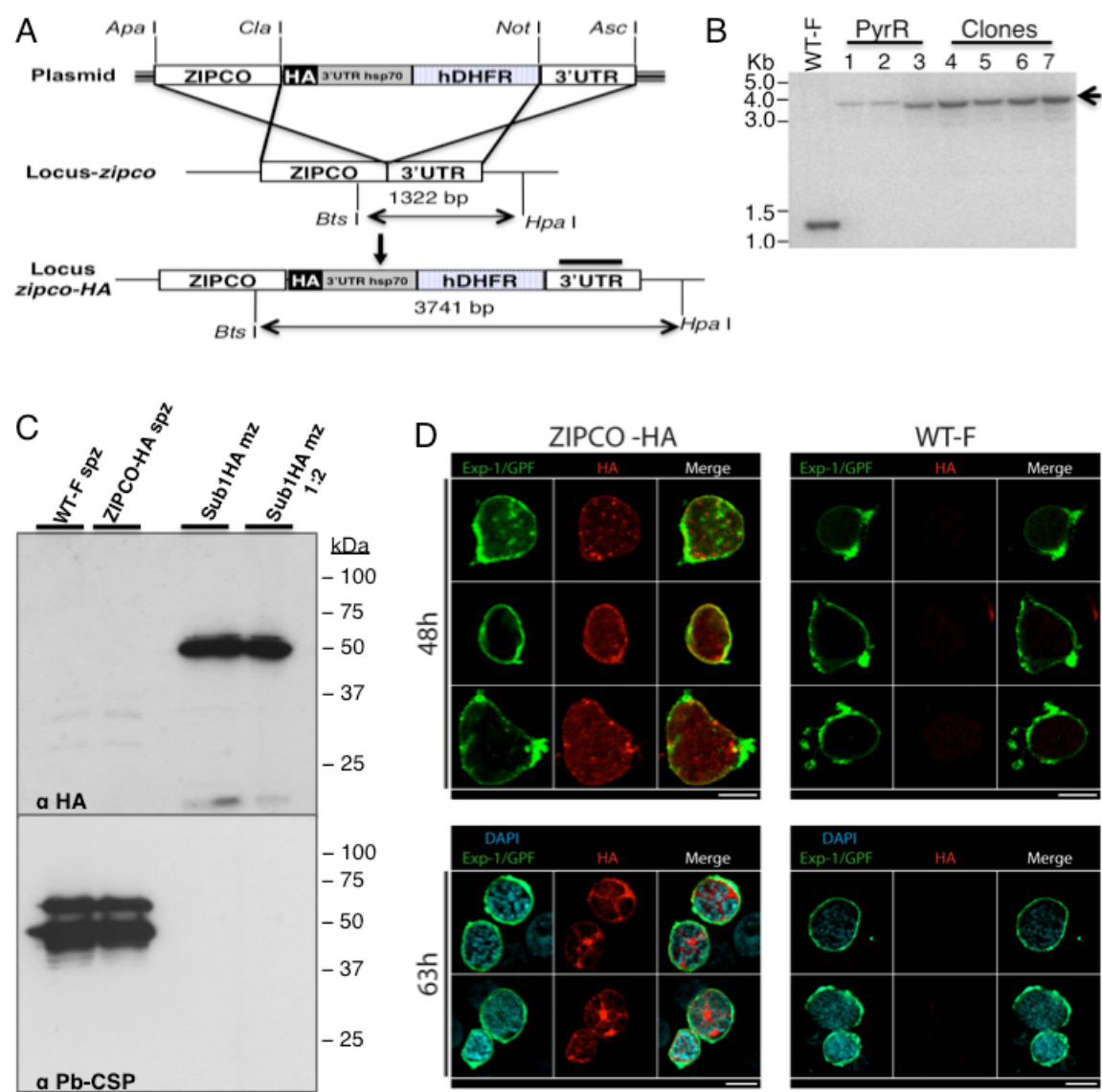

**Figure S5. Generation of ZIPCO-HA parasites and expression of ZIPCO-HA**

Supplement: Supplementary file 7 [file emmm0006-1387-sd7.pdf]

**Figure S6. Effect of Zinc, Iron and DFO on growth of WT-F and ZIPCO-F EEFs**

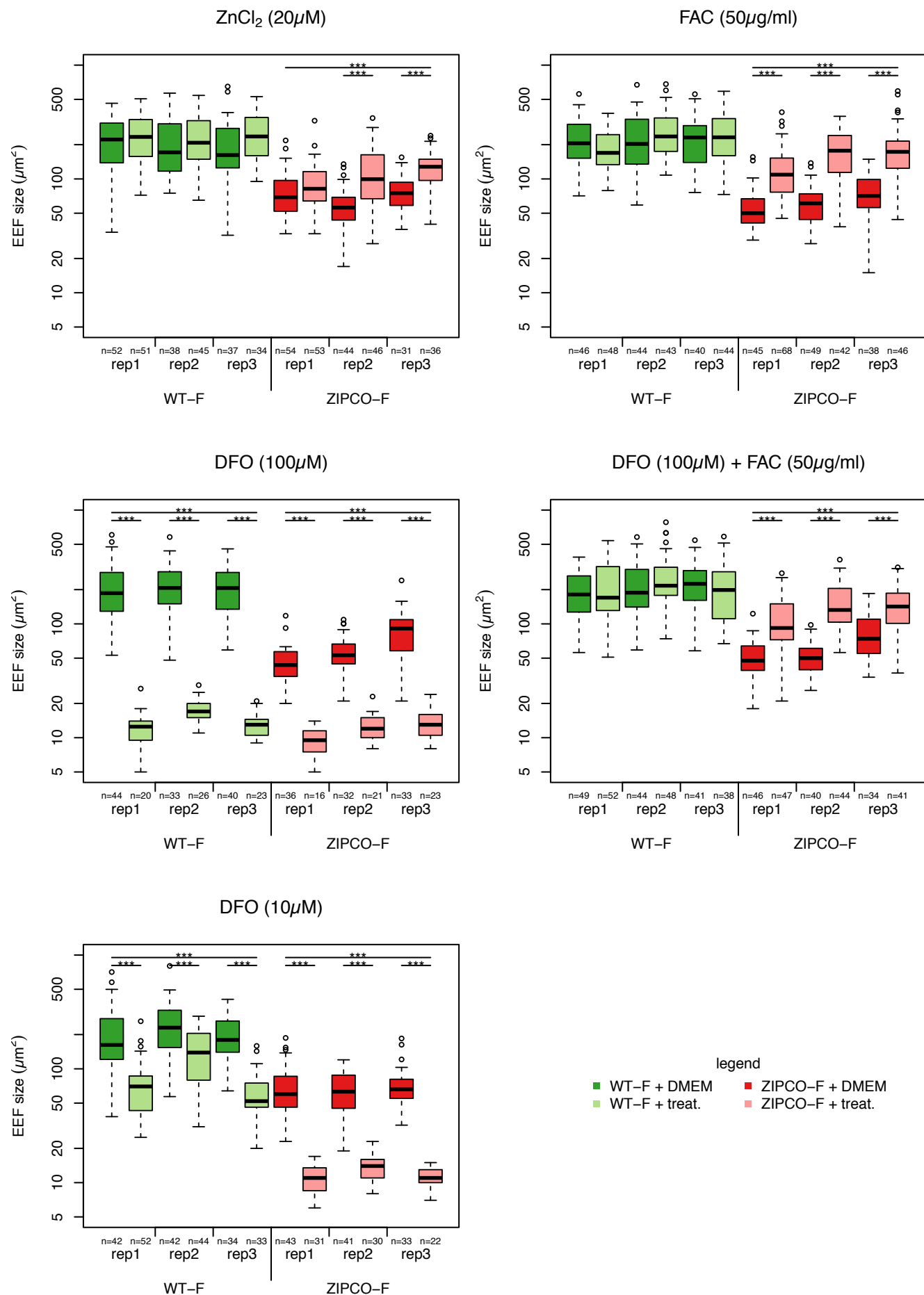

Supplement: Supplementary file 8 [file emmm0006-1387-sd8.pdf]

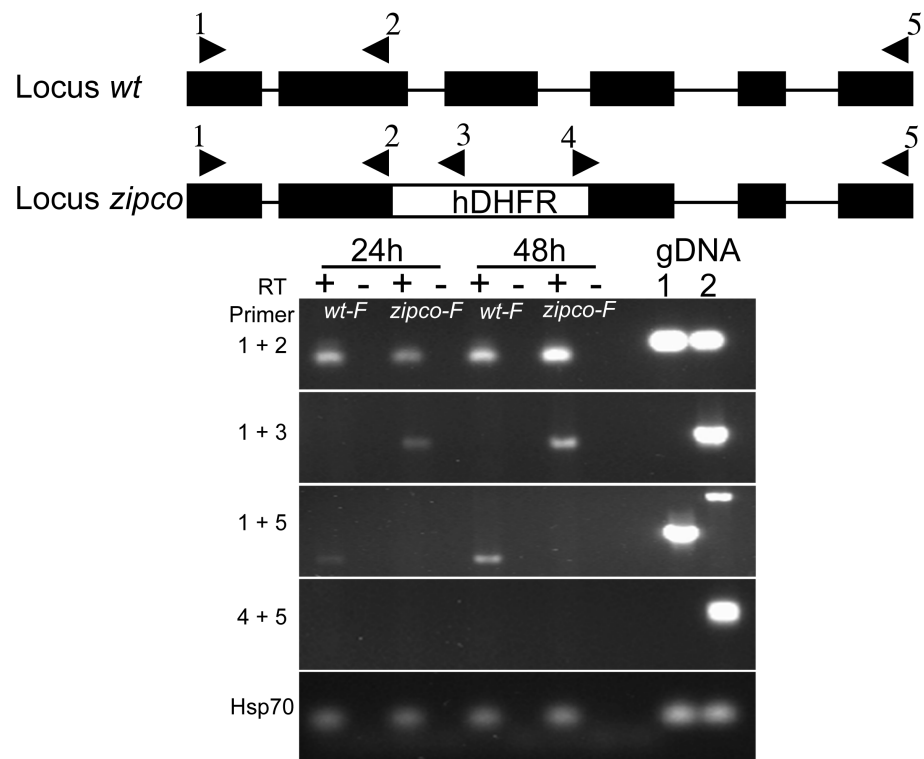

**Figure S7. RT-PCR analysis of *PBAN* *KA\_050650* transcripts in WT-F and ZIPCO-F liver stages**

Supplement: Supplementary file 9 [file emmm0006-1387-sd9.pdf]

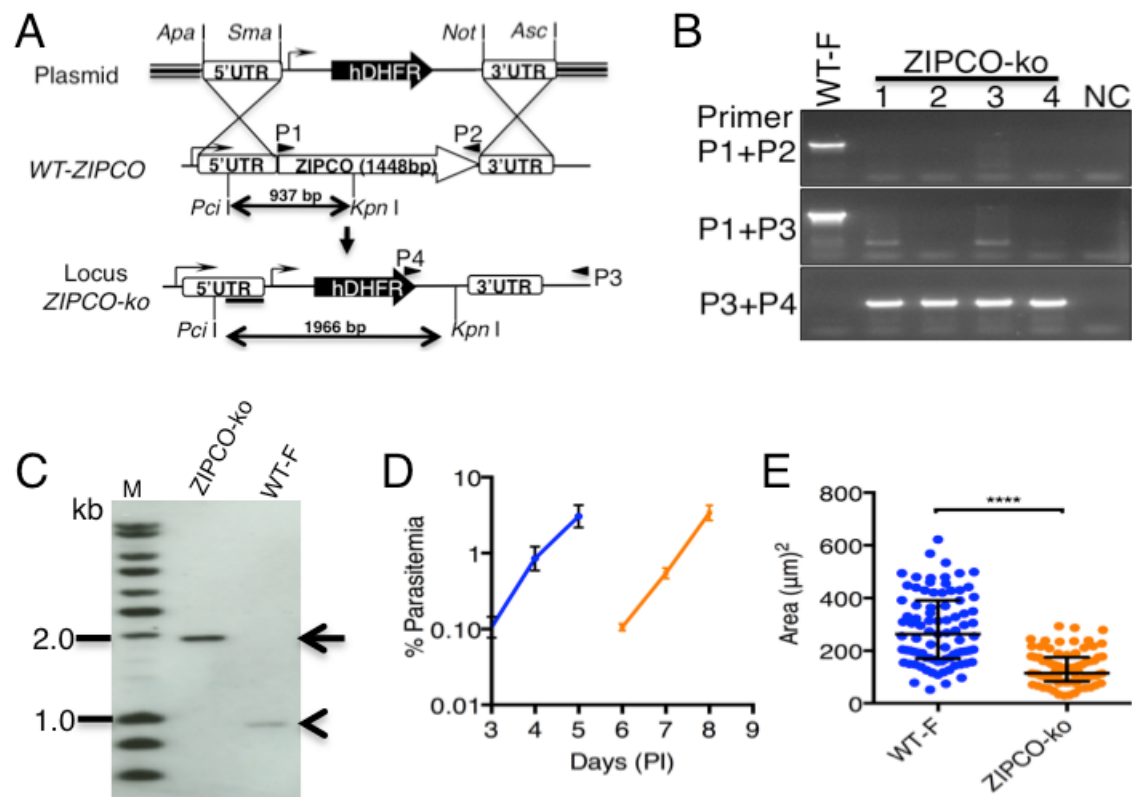

**Figure S8. Generation of ZIPCO-ko parasites, sporozoite infectivity and EEF size**

Supplement: Supplementary file 10 [file emmm0006-1387-sd10.pdf]
