# Supplementary material for: ZIPCO, a putative metal ion transporter, is crucial for Plasmodium liver-stage development
Source: EMBO Mol Med. 2014 Sep 25;6(11):1387–97. doi: 10.15252/emmm.201403868 (PMC4237467; doi:10.15252/emmm.201403868)
Supplement: Supplementary file 11 [file emmm0006-1387-sd11.pdf]

Table S1 : Effect of mutation on infectivity to mosquito. No significant difference was observed between wild type and mutant.

**Parasite**	**Number infections**	**Average percentage of females with sporozoites in Salivary glands (range)**	**Average number Salivary gland sporozoites per female x 10^3 (range)**
WT-F	16	52 (11-92)	26.7 (12-57)
ZIPCO-F	11	54 (20-84)	30.2 (15-75)
ZIPCO-HA	3	48 (17-68)	35.7 (12-59)
WT	7	Not applicable	23.7 (10-59)
ZIPCO	5	Not applicable	12.0 (9-20)
ZIPCO-ko	1	55	15.7
