# Supplementary material for: ZIPCO, a putative metal ion transporter, is crucial for Plasmodium liver-stage development
Source: EMBO Mol Med. 2014 Sep 25;6(11):1387–97. doi: 10.15252/emmm.201403868 (PMC4237467; doi:10.15252/emmm.201403868)
Supplement: Supplementary file 12 [file emmm0006-1387-sd12.pdf]

Table S2. Delay in blood stage parasitemia after intravenous injection of sporozoites.

A. ZIPCO compared to NK65 (WT) sporozoites.

**Animals**	**Parasite**	**Prepatent period**	**Average Parasitemia (day; range)**	**Nb Infected/total**	**Mode Transmission**
RAT	WT	D3	4.0 (D6 ; 1.1- 6.50)	4/4	IV-10000 spz
RAT	ZIPCO	D5	0.03 (D6 ; 0.03- 0.04)	4/4	IV-10000 spz
C57Bl6	WT	D4	1.60 (D7 ; 0.5- 3.10)	5/5	IV-30000 spz
C57Bl6	ZIPCO	D7	<0.01 (D7)	2/5 (D7)	IV-30000 spz

B. ZIPCO-HA and ZIPCO-ko compared to WT-F sporozoites

Animals	Parasite	Prepatent Period	Average Parasitemia (day ; range)	Nb Infected/total	Mode Transmission
C57Bl6	WT-F	D3	2.75 (D5 ; 1.1- 4.68)	6/6	IV 30000 spz
C57Bl6	ZIPCO-ko	D5	0.92 (D7 ; 0.4- 2.78)	6/6	IV 30000 spz
C57Bl6	WT-F	D3	2.88 (D5 ; 1.17- 5.92)	7/7	IV 25000 spz
C57Bl6	ZIPCO-HA	D3	3.03 (D5 ; 1.7- 4.86)	7/7	IV 25000 spz
