# Supplementary material for: ZIPCO, a putative metal ion transporter, is crucial for Plasmodium liver-stage development
Source: EMBO Mol Med. 2014 Sep 25;6(11):1387–97. doi: 10.15252/emmm.201403868 (PMC4237467; doi:10.15252/emmm.201403868)
Supplement: Supplementary file 13 [file emmm0006-1387-sd13.pdf]

Table S3: Primers used in the study

S#	Name	Primer Sequence	Used for	Described/shown in
**Primers for qRT-PCR of *zipco* expression**				
1	qPCR-F	5'-TAATAGTTGGCCTCTCGATG-3'	Quantification of *zipco* mRNA	Figure E1
2	qPCR-R	5'-ACGATAGCACTGCAACAAG-3'	Quantification of *zipco* mRNA	Figure E1
**Primers for ZIPCO gene disruption and verification of recombinant clones**				
3	ApaI	5'-**GGGCCC**CAACATTTTTATCAACAACGGAC-3'	Plasmid construct ZIPCO and ZIPCO-HA	Figure 2A; Figure E5A
4	SmaI	5'-**CCCGGG**GAAAGGTTCGTATTTTCTTTAATGCTTAATG-3'	Plasmid construct ZIPCO	Figure 2A
5	NotI	5'-**GCGGCCGC**CCTTACCATTAGGAATTCTTGTTGCAG-3'	Plasmid construct ZIPCO	Figure 2A
6	AscI	5'**GGCGCGCC**AAGTATTATATTTCGCACGGATGATAGAAC-3'	Plasmid construct ZIPCO	Figure 2A
7	1	5'-CGC**GGATCC**ATGTGGCTAAAACTGATTCTGGCTATA-3'	RT-PCR analysis transcripts	Primer 1 Figure E7
8	2	5'-CATTTCTATATCTATATGGTCACGG-3'	RT-PCR analysis transcripts	Primer 2 Figure E7
9	3	5'-TGCTTTGAGGGGTGAGCATTTAAAGC-3'	RT-PCR analysis transcripts	Primer 3 Figure E7
10	4	5'-TGTTGTCTCTTCAATGATTCATAAATAGTTGG-3'	RT-PCR analysis transcripts	Primer 4 Figure E7
11	5	5'-CCG**CTCGAG**CGGCTACATTGGATTTTTCTAAATAATTAAATAC-3'	RT-PCR analysis transcripts	Primer 5 Figure E7
12	hsp70F	5'-TGCAGCAGATAATCAAACTC-3'	RT-PCR analysis transcripts; qRT-PCR	Hsp70 Figure E7
13	hsp70R	5'-ACTTCAATTTGTGGAACACC-3'	RT-PCR analysis transcripts; qRT-PCR	Hsp70 Figure E7
**Primers used for ZIPCO-HA construct**				
14	Cla-Hpa	5'-**ATCGATGTTAAC**GCATATATCAATTCATCATTAAA-3'	Plasmid construct ZIPCO-HA	Figure E5A
15	HpaI	5'-**GTTAAC**TATTAAATTTTTTTTGTAAAGC-3'	Plasmid construct ZIPCO-HA	Figure E5A

16	ClaI	5'-**ATCGAT**AACTACATTGGATTTTTCTAAATAATTAAATAC-3'	Plasmid construct ZIPCO-HA	Figure E5A
17	HA-NotI	5'-ATAAGAAT**GCGGCCGC**AATAGTCATCCCCATTTCATTC-3'	Plasmid construct ZIPCO-HA	Figure E5A
18	HA-AscI	5'-**GGCGCGCC**CCATAAAATATATAGCATTGCAC-3'	Plasmid construct ZIPCO-HA	Figure E5A
19	551	5'-GCAAGGCGATTAAGTTG-3'	Sequencing of HA-Construct	NA
20	1251	5'-GTAGAAGGTACCGCGC-3'	Sequencing of HA-Construct	NA
21	1710	5'-GGAATTCTTGTTGCAGTGCTATCG-3'	Sequencing of HA-Construct	NA
22	4420	5'-CTTCAATGATTCATAAATAGTTGGAC-3'	Sequencing of HA-Construct	NA
23	5180	5'-GTGTATCTTAAGAAGAATTGCAATTTC-3'	Sequencing of HA-Construct	NA
24	HA-Probe-F	5'-GTATTATATTTCGCACGGATGATAGAAC-3'	Probe for southern blot	Figure E5A
25	HA-Probe-R	5'-CTTACCATTACCATTAGGAATTCTTGTTGCAG-3'	Probe for southern blot	Figure E5A
**Primers for ZIPCO-ko construct**				
26	DApaI	5'-**GGGCCC**GAAGTGTTATAATATATTTAATAG-3'	Plasmid construct ZIPCO-ko	Figure E8A
27	DSmaI	5'-TCC**CCCGGG**TTTAATAAAATATATAATATTC-3'	Plasmid construct ZIPCO-ko	Figure E8A
28	DNotI	5'-ATAAGAAT**GCGGCCGC**AGTCATCCCCATTTCATTCAAATG-3'	Plasmid construct ZIPCO-ko	Figure E8A
29	DAscI	5'-**GGCGCGCC**TTGCAATTCTTCTTAAGATACACTT-3'	Plasmid construct ZIPCO-ko	Figure E8A
30	P1	5'-ATGTGGCTAAAACTGATTCTGG-3'	PCR Verification of KO-clone	Figure E8A
31	P2	5'-TATCATTAATGTTGACATCCCAC-3'	PCR Verification of KO-clone	Figure E8A
32	P3	5'-CTATTGCTTAAGTTTATGCGAATAC-3'	PCR Verification of KO-clone	Figure E8A
33	P4	5'-GTGCATGCACATGCATGTAAATAG-3'	PCR Verification of KO-clone	Figure E8A

Restriction sites are highlighted in bold letters.
