# Supplementary material for: ZIPCO, a putative metal ion transporter, is crucial for Plasmodium liver-stage development
Source: EMBO Mol Med. 2014 Sep 25;6(11):1387–97. doi: 10.15252/emmm.201403868 (PMC4237467; doi:10.15252/emmm.201403868)
Supplement: Supplementary file 14 [file emmm0006-1387-sd14.pdf]

Table S4: **statistical analysis of the effect of Zinc and Iron on EEF size.** Analysis was conducted within treatments across replicates using a non- parametric test, followed by combination of P values from independent tests of significance using the meta- analytical approach of Fisher (Fisher, 1932). Briefly, each treatment condition was compared to the control condition (DMEM) for each biological replicates independently, using the exact two sided Wilcoxon rank sum test. Then, the P values for each treatment were combined using Fisher’s method. The threshold for significance was defined as p= 0.001. All computations were done using the R statistical software (version 2.14.1; R Development Core Team, 2011) and the exactRankTests package (version 0.8- 22; Hothorn and Hornik, 2011).

				EEF	EEF size					Fisher's combined probability		
**experiment**	**condition**	**parasite**	**replicate**	**#**	**median**	**mean**	**sd**	**Z-statistic**	**p-value**	**chi2**	**df**	**p-value**
ZnCl2_20	DMEM	WT-F	rep1	52	222	232.3	110.1	-0.64	0.52			
ZnCl2_20	ZnCl2_20	WT-F	rep1	51	234	244.8	106.4					
ZnCl2_20	DMEM	WT-F	rep2	38	171	209.2	114.6	-1.5	0.15	10.61	6	0.1
ZnCl2_20	ZnCl2_20	WT-F	rep2	45	208	241.1	117.2					
ZnCl2_20	DMEM	WT-F	rep3	37	162	207.3	134.4	-1.8	0.065			
ZnCl2_20	ZnCl2_20	WT-F	rep3	34	236	250.8	116.1					
ZnCl2_20	DMEM	ZIPCO-F	rep1	54	69	80.5	38.6	-1.8	0.08			
ZnCl2_20	ZnCl2_20	ZIPCO-F	rep1	53	82	94.1	49.3					
ZnCl2_20	DMEM	ZIPCO-F	rep2	44	56	58.6	25.5	-5.1	9.1e-08	63.93	6	7.1e-12
ZnCl2_20	ZnCl2_20	ZIPCO-F	rep2	46	99.5	121	70.7					
ZnCl2_20	DMEM	ZIPCO-F	rep3	31	75	79.1	29	-4.6	1.8e-06			
ZnCl2_20	ZnCl2_20	ZIPCO-F	rep3	36	128	129.4	47.9					
FAC50	DMEM	WT-F	rep1	46	205.5	230.9	113.9	1.8	0.078			
FAC50	FAC50	WT-F	rep1	48	169.5	187.3	77.1					
FAC50	DMEM	WT-F	rep2	44	203	243.5	136.1	-1.3	0.19	10.03	6	0.12
FAC50	FAC50	WT-F	rep2	43	236	271.7	129.4					
FAC50	DMEM	WT-F	rep3	40	231.5	236.3	112.7	-0.77	0.44			
FAC50	FAC50	WT-F	rep3	44	232	265.9	135.1					
FAC50	DMEM	ZIPCO-F	rep1	45	50	59.7	27.3	-6.5	3.9e-12			
FAC50	FAC50	ZIPCO-F	rep1	68	109	124.4	67.4					
FAC50	DMEM	ZIPCO-F	rep2	49	61	63.6	24.9	-7.3	1.1e-16	179.9	6	3.6e-36
FAC50	FAC50	ZIPCO-F	rep2	42	177	178.9	76.3					
FAC50	DMEM	ZIPCO-F	rep3	38	71	76.1	32.3	-6.4	2,00E-12			
FAC50	FAC50	ZIPCO-F	rep3	46	173	193.7	113.2					
DFO100	DMEM	WT-F	rep1	44	186	217.5	126.2	6.4	5.1e-17			
DFO100	DFO100	WT-F	rep1	20	12.5	12.3	4.9					
DFO100	DMEM	WT-F	rep2	33	207	225	109.4	6.6	5.1e-17	226.9	6	3.6e-46
DFO100	DFO100	WT-F	rep2	26	17	17.6	3.9					
DFO100	DMEM	WT-F	rep3	40	206.5	211.2	104.6	6.6	2.1e-17			
DFO100	DFO100	WT-F	rep3	23	13	13.3	3.4					
DFO100	DMEM	ZIPCO-F	rep1	36	43.5	46.6	18.9	5.7	9.6e-14			
DFO100	DFO100	ZIPCO-F	rep1	16	9.5	9.3	2.7					
DFO100	DMEM	ZIPCO-F	rep2	32	53	58.5	21.9	6.1	9.4e-15	191.8	6	1.1e-38
DFO100	DFO100	ZIPCO-F	rep2	21	12	12.5	3.5					
DFO100	DMEM	ZIPCO-F	rep3	33	91	91	44.5					
DFO100	DFO100	ZIPCO-F	rep3	23	13	13.9	4.3	6.3	2.5e-15			
DFO100FAC50	DMEM	WT-F	rep1	49	181	197.3	90.8	-0.6	0.55			
DFO100FAC50	DFO100_FAC50	WT-F	rep1	52	170	219.8	126.5					
DFO100FAC50	DMEM	WT-F	rep2	44	188	227	125.5	-1.4	0.17	6.946	6	0.33
DFO100FAC50	DFO100_FAC50	WT-F	rep2	48	216.5	259	146.8					
DFO100FAC50	DMEM	WT-F	rep3	41	225	238.8	109.8	0.97	0.33			
DFO100FAC50	DFO100_FAC50	WT-F	rep3	38	198.5	220.4	122.1					
DFO100FAC50	DMEM	ZIPCO-F	rep1	46	47.5	53.2	19.9	-6	1.9e-10			
DFO100FAC50	DFO100_FAC50	ZIPCO-F	rep1	47	92	112.7	60					
DFO100FAC50	DMEM	ZIPCO-F	rep2	40	50	51.7	16.8	-7.4	6.5e-18	155.2	6	6E-31
DFO100FAC50	DFO100_FAC50	ZIPCO-F	rep2	44	132.5	156.9	77.6					
DFO100FAC50	DMEM	ZIPCO-F	rep3	34	74	81.5	36.1	-5	1.6e-07			
DFO100FAC50	DFO100_FAC50	ZIPCO-F	rep3	41	142	151.6	66					
DFO10	DMEM	WT-F	rep1	42	162	218	147.2	6.2	2.2e-11			
DFO10	DFO10	WT-F	rep1	52	70	76.8	43.8					
DFO10	DMEM	WT-F	rep2	42	230	254	142.4	4.1	2.8e-05	126.5	6	7E-25
DFO10	DFO10	WT-F	rep2	44	139	144.3	68.1					
DFO10	DMEM	WT-F	rep3	34	179.5	192.1	80.7	6.3	5.5e-13			
DFO10	DFO10	WT-F	rep3	33	52	62.7	31.2					
DFO10	DMEM	ZIPCO-F	rep1	43	60	70.6	37	7.3	3,00E-21			
DFO10	DFO10	ZIPCO-F	rep1	31	11	11.2	3					
DFO10	DMEM	ZIPCO-F	rep2	41	63	63.8	26.6	7.1	3.4e-19	247.8	6	1.2e-50
DFO10	DFO10	ZIPCO-F	rep2	30	14	14.1	3.8					
DFO10	DMEM	ZIPCO-F	rep3	33	66	74.5	33.1	6.2	1.5e-15			
DFO10	DFO10	ZIPCO-F	rep3	22	11	11	2.4					
