# Supplementary material for: ZIPCO, a putative metal ion transporter, is crucial for Plasmodium liver-stage development
Source: EMBO Mol Med. 2014 Sep 25;6(11):1387–97. doi: 10.15252/emmm.201403868 (PMC4237467; doi:10.15252/emmm.201403868)
Supplement: Supplementary file 15 [file emmm0006-1387-sd15.pdf]

**Supplementary Information Figure Legends**

**Figure S1. Expression of *PBANKA_050650* mRNA during the life cycle.**
Dot plot representation of *PBANKA_050650* relative gene expression in *P. berghei* mixed blood stages (mixed RBC), salivary gland sporozoites (spz) and HepG2 cells 5, 17 and 50 h post infection (LS) as determined by quantitative real time PCR. The values were normalized relative to the expression of *hsp70* mRNA in each sample. Means with SEM. *** indicates statistically significant ($p = 0.0078$) using one-way analysis of variance (Prism software) N=2.

**Figure S2. PBANKA_050650 contains a conserved ZIP domain.**
The PBANKA_050650 contains a ZIP domain (Pfam02535) and its sequence was aligned on the forty sequences constituting the seed of the Pfam02535 profile using Hmmer 3.0. It shows that the well conserved residues of the hmm profile are also conserved in PBANKA_050650 and its orthologues in other *Plasmodium* species. Based on this alignment, a phylogenetic tree was generated (see Figure 1B in main text) using the neighbour-joining method with 1000 bootstraps (MEGAS 4.0). The alignment presented is in the region of the ZIP domain (shown as a red box).

**Figure S3. ZIPCO-F sporozoites have normal gliding motility and cell traversal activity.**
A. ZIPCO-F sporozoites glide normally on glass slide.
Merged images of ZIPCO-F sporozoites (Green) stained with anti-CSP conjugated to Alexa-568 fluorophore (Red).
B. Flow cytometric analysis of cell traversal by sporozoites. Representative dot plots showing rhodamine-dextran positive cell populations in non-infected HepG2 cells (Control), non-infected scratched cells (Scratched), HepG2 cells infected with WT-F or ZIPCO-F sporozoites.

**Figure S4. ZIPCO parasites are defective in liver stage development.**
A. Representative image of WT and ZIPCO EEFs *in vitro*.
Cells were infected with WT (top panel) or ZIPCO sporozoites (lower panel) and after 24, 48 and 67 h the samples were fixed and stained with either a mouse monoclonal anti-CS (24 h) or a chicken anti-EXP1 antibody (48 and 67 h) and detected with a secondary antibody-AF568. Images were acquired on Zeiss epiflourescence microscope and analysed by ImageJ software. Scale bar represents 10 μM.
B. Representative micrographs of WT-F and ZIPCO-F EEFs showing nuclei.
EEFs developing *in vitro* were stained with DAPI at 53 hpi. Images were acquired on Zeiss epiflourescence microscope and analysed by ImageJ software. Scale bar represents 10 μM.

**Figure S5. Generation of ZIPCO-HA parasites and expression of ZIPCO-HA.**
A. The plasmid pBC-*zipco-HA*-hDHFR (shown on the top) contains a HA-FLAG tag fused in frame to the carboxy terminus of the ZIPCO coding sequence, followed by the 3'UTR of *Pbhsp70* and the hDHFR cassette. The targeted *Pbzipco* locus is shown in middle and the recombinant locus, *zipco-HA*, resulting from homologous recombination

on the bottom. The wild type locus is as described in figure 2A. A black bar indicates the probe used for Southern blot analysis.

B. Southern blot analysis of ZIPCO-HA recombinant parasites.

Genomic DNAs of WT-F and ZIPCO-HA parasite populations obtained after transfection and pyrimethamine drug selection (lanes 1-3) and four ZIPCO-HA clones (lanes 4-7) were digested with *Bts*1 and *Hpa*1. WT (1286 bp) and recombinant (3741 bp) restriction fragments are indicated by double-headed arrows.

C. Western blot analysis of ZIPCO-HA sporozoites

Protein extracts from 6 x $10^5$ WT-F and 6 x $10^5$ ZIPCO- HA sporozoites were denatured in Laemmli buffer (Biorad) supplemented with 5% β-mercaptoethanol, separated on a SDS acrylamide gel and transferred to a nitrocellulose membrane. The membrane was incubated with a monoclonal anti-HA antibody (1: 500; Roche), followed by a goat anti rat HRP antibody (1:10000). Revelation of bands was performed with ECL Western Blotting Substrate (Pierce) and Amersham Hyperfilm ECL (GE Healthcare). Positive controls for detecting the HA tag are 2 x $10^6$ and 1 x $10^6$ merozoites of a *P. berghei* Subtilisin 1-HA tagged recombinant line (kindly provided by Dr J-C. Barale prior to publication) (see top panel, α HA). The filter was then stripped and incubated with a mouse monoclonal anti-CS IgG antibody (1:5000; Yoshida et al., 1980) followed by secondary anti-mouse IgG HRP conjugate 1:1000 (Promega) (bottom panel, α Pb CSP). These results show that ZIPCO-HA is not detectable at the sporozoite stage.

D. Expression of ZIPCO-HA in Liver stages.

HepG2 cells were infected with WT-F or ZIPCO-HA sporozoites and samples were fixed with 4% PFA at 48 and 63 h post-infection. Samples were incubated with rat monoclonal anti-HA (Roche) and chicken anti-EXP1 antibodies, followed by Alexa Fluor 568 and 647 conjugated antibodies, then stained with DAPI and mounted in ProLong® Antifade Kit (Molecular probes). 14-bit images were acquired at the Imaging Platform of the Pasteur Institute (PFID) using a spinning-disk confocal system (UltraView ERS, Perkin Elmer). Images were acquired using a 63X oil objective (Zeiss) and covering a distance of 3 to 5.5 µm along the Z-axis. Images were processed using the ImageJ software (Schneider et al, 2012). A specific signal was detected in EEFs at 48 and 63 hpi. At 48 hpi, a weak but specific signal was detected, as a punctuated labelling within and at the periphery of the developing parasite (top panel). Double labelling with EXP-1, a parasite protein located in the parasitophorous vacuole membrane (PVM), showed only a few discrete regions of overlap with the HA signal. At 63 hpi, the HA labelling was detected around groups of nuclei, segmenting the developing EEF (lower panel). This labelling is very similar to that obtained with proteins present in the parasite plasma membrane at the cytomere stage (Graewe et al, 2011). These data, together with the images obtained with MSP1 (main text Fig 4) strongly suggest that ZIPCO-HA is mainly located at the parasite plasma membrane.

**Figure S6. Effect of Zinc, Iron and DFO on growth of WT-F and ZIPCO-F EEFs**

Two hours post-infection with sporozoites, media was removed and replaced with DMEM or DMEM supplemented with $ZnCl_2$, FAC or DFO at the concentrations indicated. The size of EEFs was measured 44h later as described in Material and Methods. Three independent experiments were performed (rep 1-3) and n indicates the

number of EEFs measured. *** indicates p <0.001. The complete statistical analysis with p values is shown in Table S4.

**Figure S7. RT-PCR analysis of *PBANKA_050650* transcripts in WT-F and ZIPCO-F liver stages.**
Top: Schematic representations of *wt* and *zipco* loci and the position of primers used for RT-PCR analysis (for primer sequences see Table S3).
Bottom: RT-PCR performed on total RNA extracted from HepG2 cells 24h and 48h post-infection with salivary gland sporozoites. (+) reverse transcriptase in reaction; (-) absence of reverse transcriptase in reaction. gDNA corresponds to PCR on genomic DNA of WT-F (1) and ZIPCO1-F (2). Note that a complete transcript of *PBANKA_050650* is only detected in WT samples (primers 1+5).

**Figure S8. Generation of ZIPCO-ko parasites, sporozoite infectivity and EEF size.**
A. Schematic representation of strategy used to obtain ZIPCO-ko parasites.
The upper part shows the plasmid used for targeting the *zipco* locus shown in the middle part. The lower part shows the recombinant *zipco-ko* locus obtained after recombination by double cross over. Primers used for genotyping the recombinant parasites are indicated by arrowheads. The probe used for Southern analysis (see panel C) is shown as a black bar below the 5'UTR region. The size of the expected fragments, both in WT and ZIPCO-ko, generated by *Pci* I and *Kpn* I are shown by double-headed arrow.
B. PCR analysis of ZIPCO-ko recombinant parasites.
PCR performed on genomic DNA extracted from WT-F and four ZIPCO-ko clones (Lane 1 to 4). Primer positions are indicated by arrowheads in panel A. NC: Negative control where PCR was performed without any DNA template.
C. Southern blot analysis of ZIPCO-ko clone.
Genomic DNA of WT-F and ZIPCO-ko clones was digested with *Pci* I and *Kpn* I. The size of the expected fragments for ZIPCO-ko (1966 bp), and WT (937 bp) are shown by an arrow and arrow-head respectively.
D. Infectivity of ZIPCO-ko sporozoites.
Graph showing the blood stage parasitemia obtained after intravenous injection with 30000 WT-F (shown in blue) or ZIPCO-ko (shown in orange) sporozoites. Data presented as mean ± SD of 5 mice in each group. Note the delay of three days after injection of ZIPCO-ko compared to WT-F spozozoites.
E. Size of WT-F and ZIPCO-ko 48 h EEFs.
The dot plot shows the median and interquartile range of the size (area) of WT-F and ZIPCO-ko EEFs. ****p <0.0001. N = 86 for WT-F and 80 for ZIPCO-ko.
