# Supplementary material for: ZIPCO, a putative metal ion transporter, is crucial for Plasmodium liver-stage development
Source: EMBO Mol Med. 2014 Sep 25;6(11):1387–97. doi: 10.15252/emmm.201403868 (PMC4237467; doi:10.15252/emmm.201403868)
Supplement: Supplementary file 16 [file emmm0006-1387-sd16.pdf]

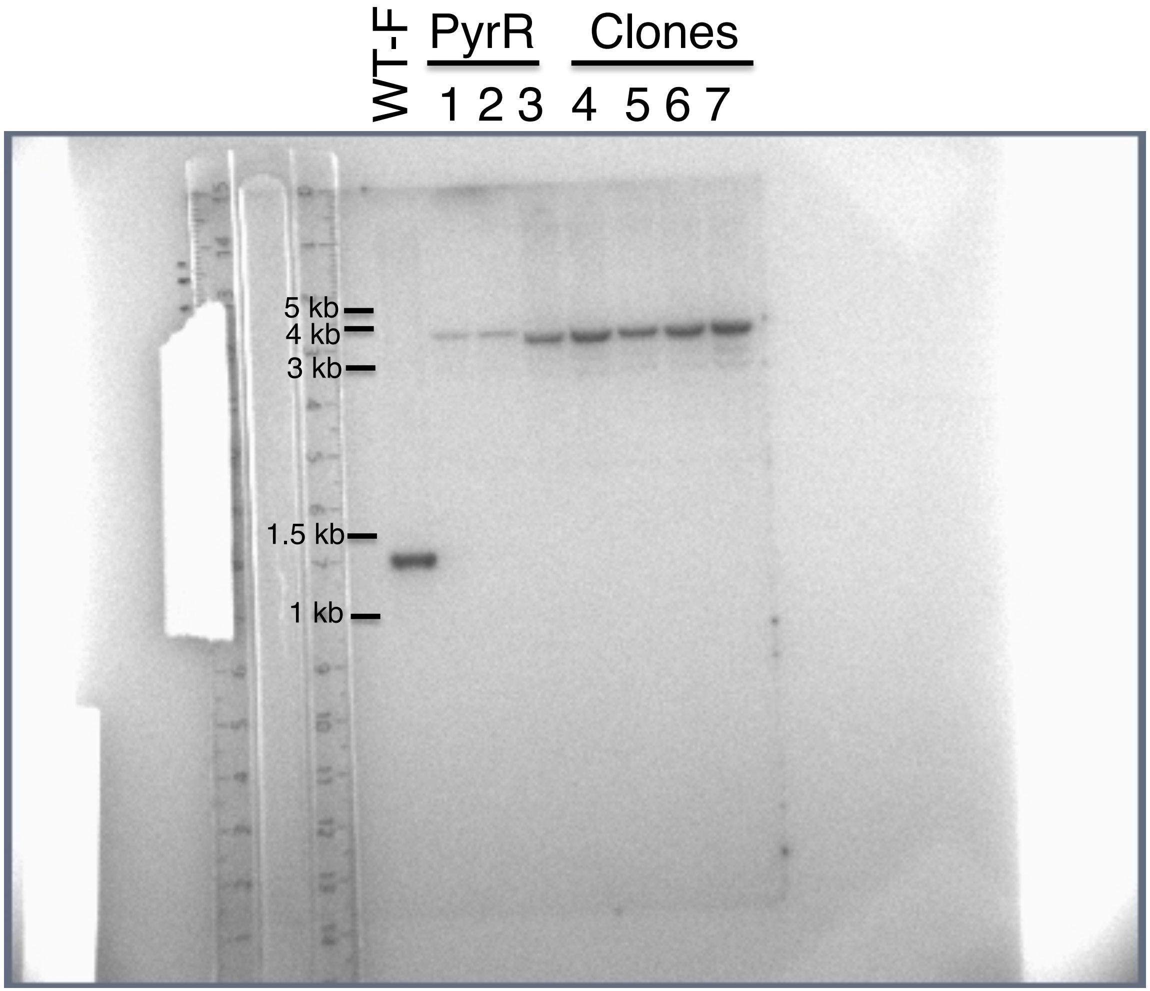

Figure S5, Panel-B: Southern blot analysis of ZIPCO-HA recombinant parasites.

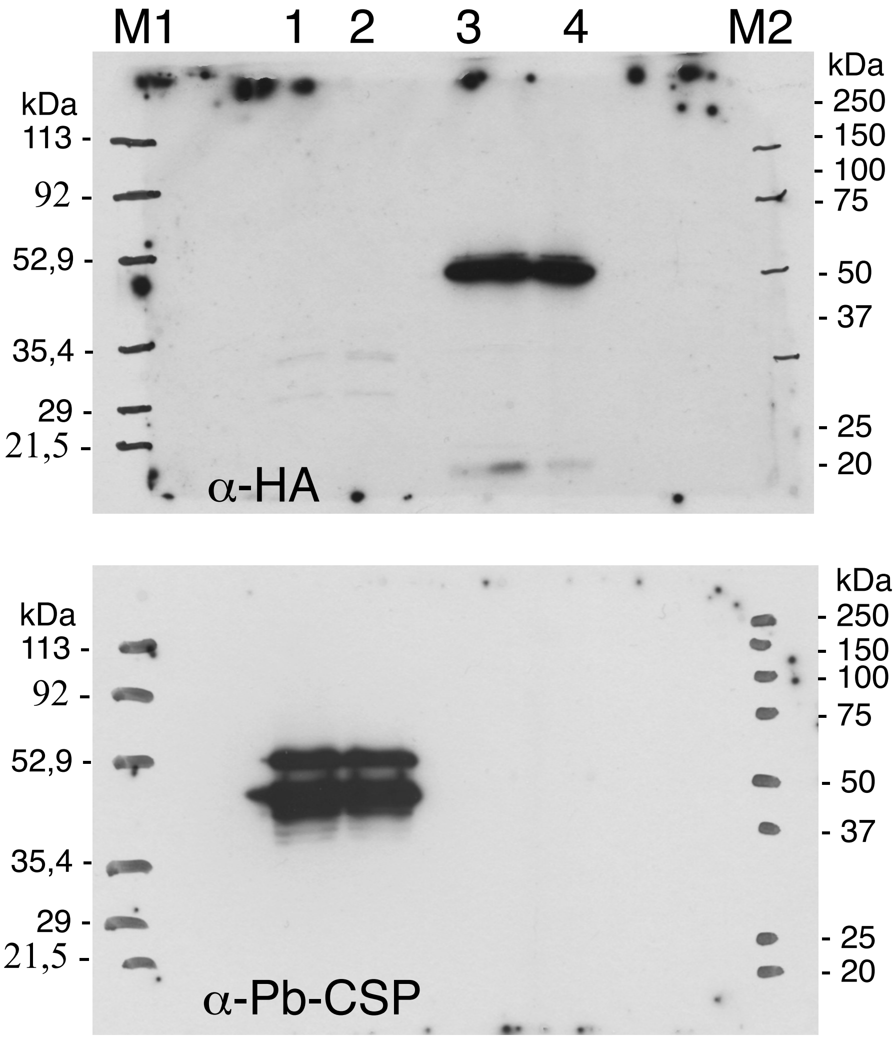

1- WT-F spz
2- ZIPCO-HA spz
3- Sub1HA mz
4- Sub1HA mz (1:2)
M- Mol Wt marker

Figure S5, Panel-C: Western blot analysis of WT-F and ZIPCO-HA sporozoites .
