# Supplementary material for: ZIPCO, a putative metal ion transporter, is crucial for Plasmodium liver-stage development
Source: EMBO Mol Med. 2014 Sep 25;6(11):1387–97. doi: 10.15252/emmm.201403868 (PMC4237467; doi:10.15252/emmm.201403868)
Supplement: Supplementary file 17 [file emmm0006-1387-sd17.pdf]

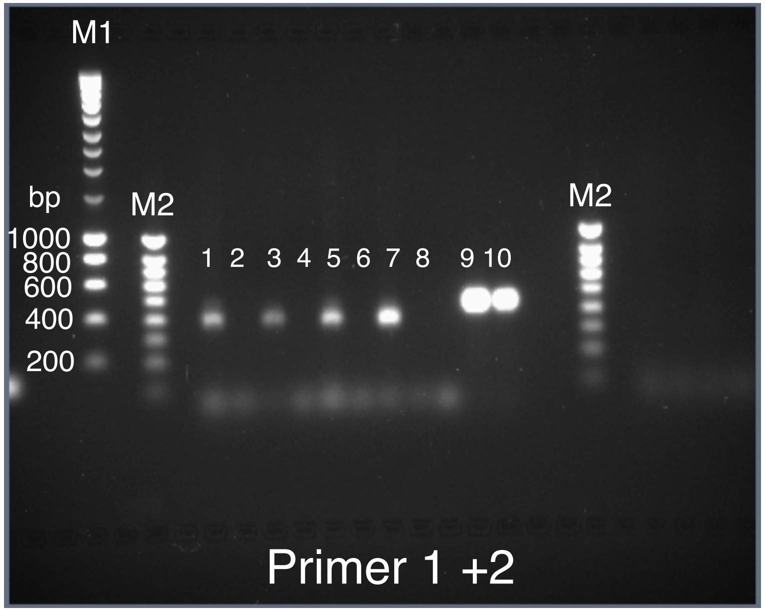

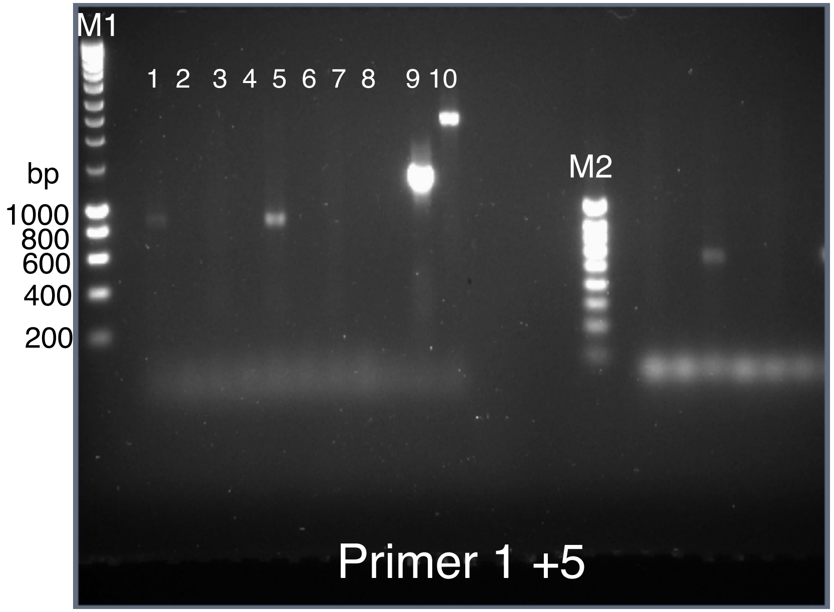

M1: Smart ladder
M2: 100bp DNA Ladder
Lane 1,2, 3 and 4: 24h Liver stage RNA
Lane 5, 6, 7 and 8: 48h Liver Stage RNA
Lane 9: Genomic DNA of WT-F as PCR template
Lane 10: Genomic DNA of ZIPCO-F as PCR template
Lane 1, 3, 5 and 7: With RT.
Lane 2, 4, 6 and 8: No RT.

Figure S7 : RT-PCR analysis of PBANKA_050650 transcripts in WT-F and ZIPCO-F liver stages (Panel 1 and 2).

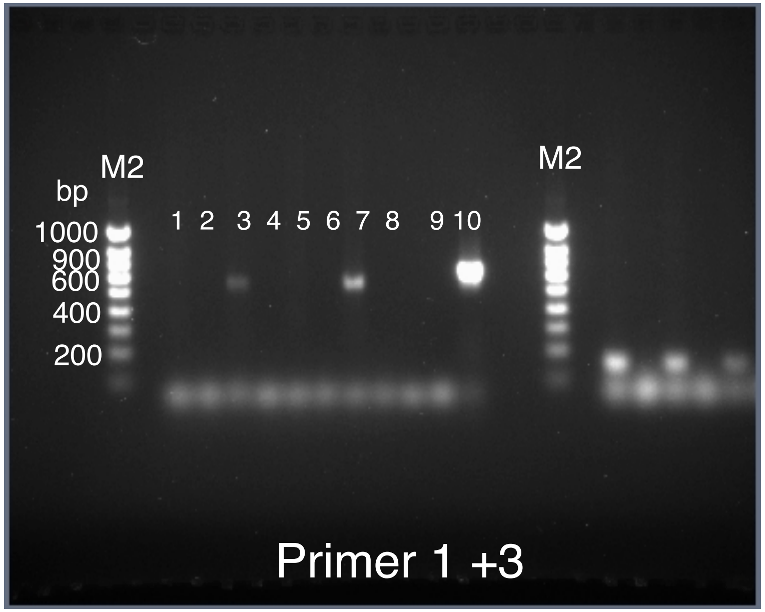

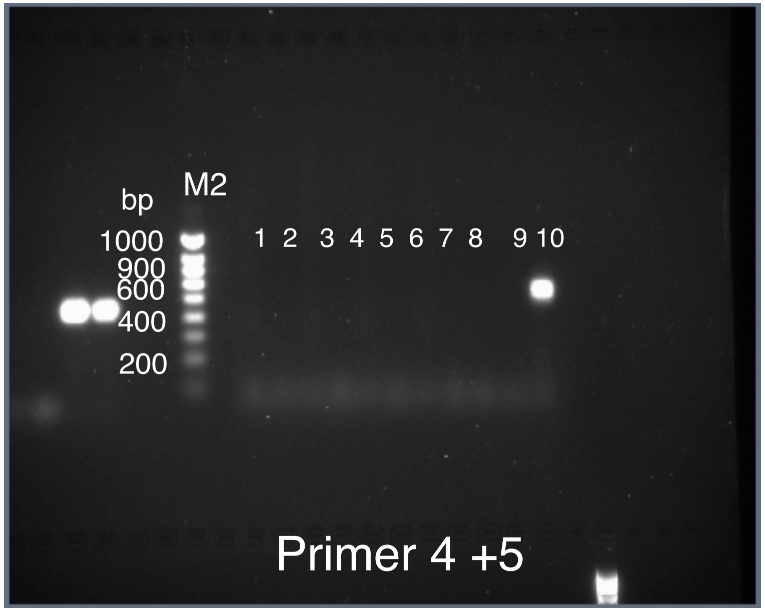

M1: Smart ladder
M2:100bp DNA Ladder
Lane 1,2, 3 and 4: 24h Liver stage RNA
Lane 5, 6, 7 and 8: 48h Liver Stage RNA
Lane 9: Genomic DNA of WT-F as PCR template
Lane 10: Genomic DNA of ZIPCO-F as PCR template
Lane 1, 3, 5 and 7: With RT.
Lane 2, 4, 6 and 8: No RT.

Figure S7 : RT-PCR analysis of PBANKA_050650 transcripts in WT-F and ZIPCO-F liver stages (Panel 3 and 4).

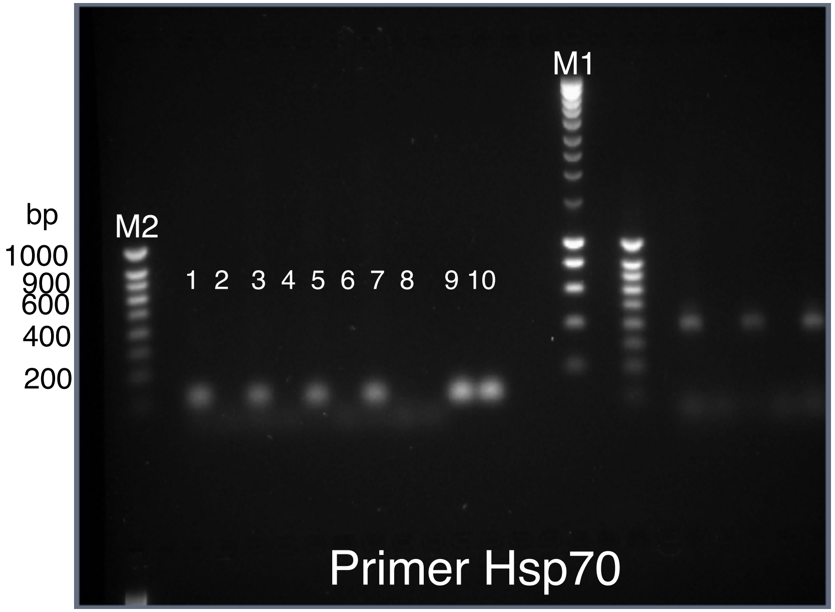

M1: Smart ladder
M2: 100bp DNA Ladder
Lane 1,2, 3 and 4: 24h Liver stage RNA
Lane 5, 6, 7 and 8: 48h Liver Stage RNA
Lane 9: Genomic DNA of WT-F as PCR template
Lane 10: Genomic DNA of ZIPCO-F as PCR template
Lane 1, 3, 5 and 7: With RT.
Lane 2, 4, 6 and 8: No RT.

Figure S7 : RT-PCR analysis of PBANKA_050650 transcripts in WT-F and ZIPCO-F liver stages (Panel 5).
