# Supplementary material for: ZIPCO, a putative metal ion transporter, is crucial for Plasmodium liver-stage development
Source: EMBO Mol Med. 2014 Sep 25;6(11):1387–97. doi: 10.15252/emmm.201403868 (PMC4237467; doi:10.15252/emmm.201403868)
Supplement: Supplementary file 18 [file emmm0006-1387-sd18.pdf]

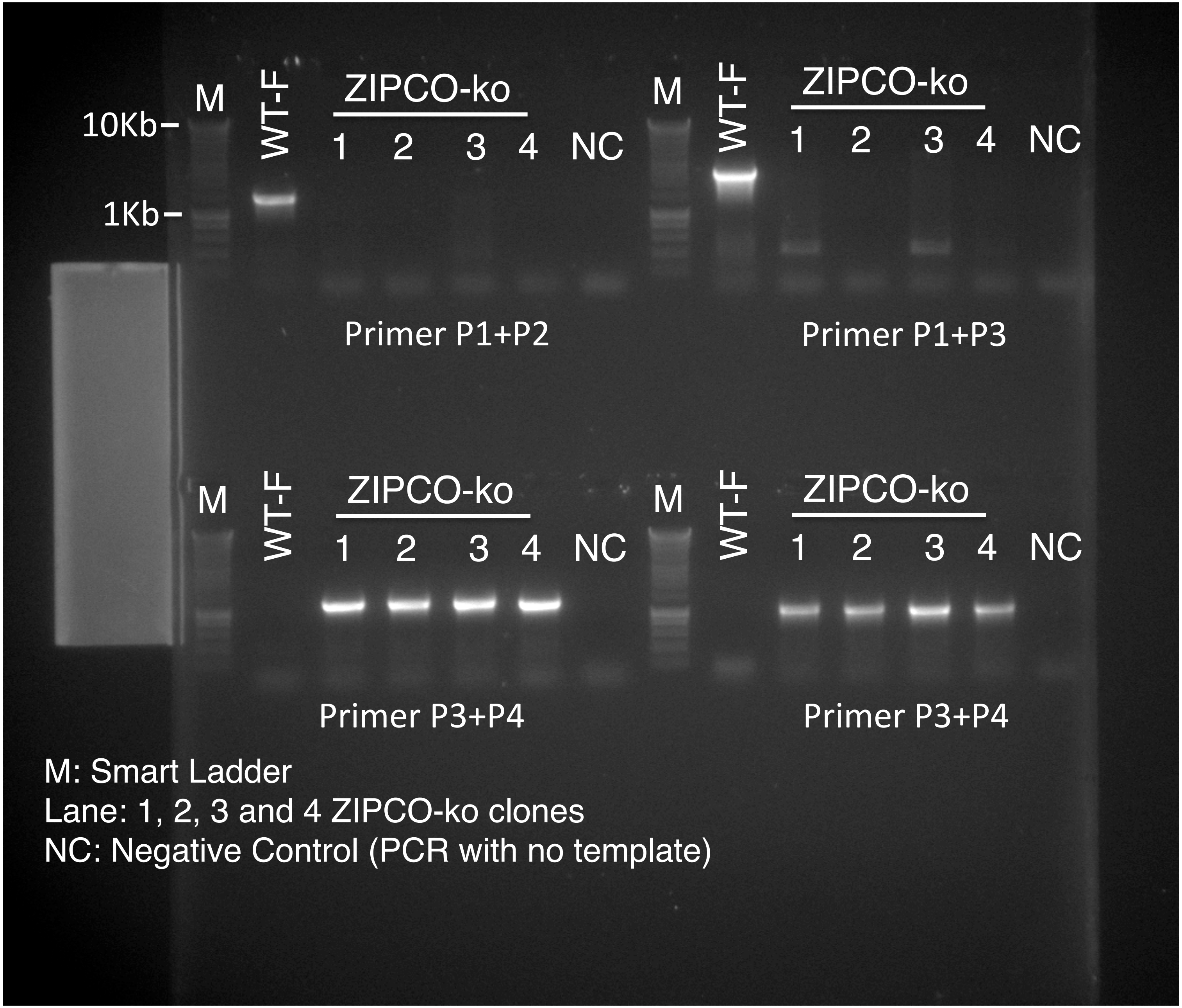

Figure S8, Panel-B : PCR analysis of ZIPCO-ko recombinant parasites.

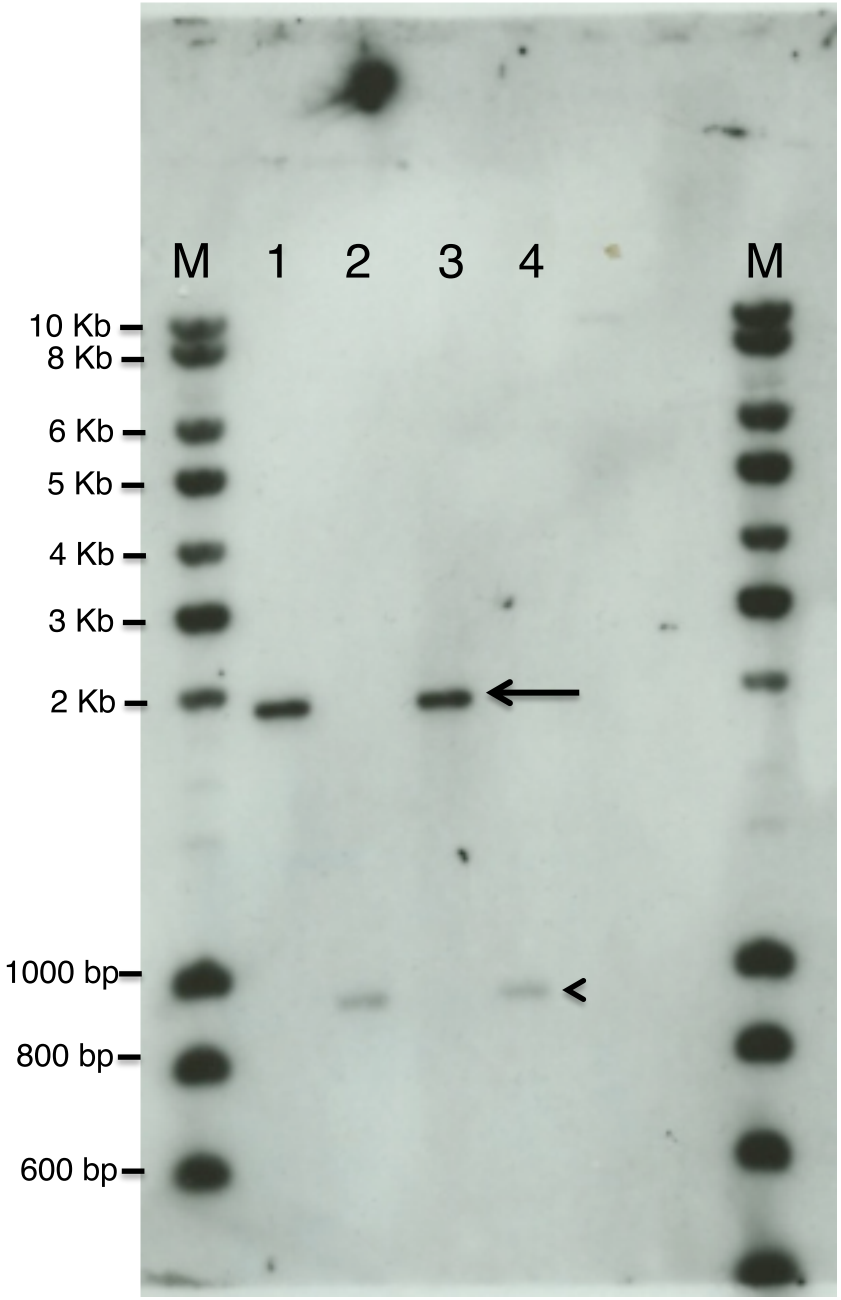

M: Molecular Wt Marker
Lane 1 and 3: ZIPCO-ko (*Pci* I + *Kpn* I digest)
Lane 2 and 4: WT-F (*Pci* I + *Kpn* I digest)

Recombinant bands shown by arrow
and WT bands by open arrow-head.

Figure S8, Panel-C : Southern Blot analysis of ZIPCO-ko clone.
